# Replicative aging is associated with loss of genetic heterogeneity from extrachromosomal circular DNA in *Saccharomyces cerevisiae*

**DOI:** 10.1093/nar/gkaa545

**Published:** 2020-07-01

**Authors:** Iñigo Prada-Luengo, Henrik D Møller, Rasmus A Henriksen, Qian Gao, Camilla Eggert Larsen, Sefa Alizadeh, Lasse Maretty, Jonathan Houseley, Birgitte Regenberg

**Affiliations:** Ecology and Evolution, Department of Biology, University of Copenhagen, Copenhagen DK-2100, Denmark; Ecology and Evolution, Department of Biology, University of Copenhagen, Copenhagen DK-2100, Denmark; Department of Biology, Institute of Biochemistry, ETH Zürich, Zurich CH-8093, Switzerland; Ecology and Evolution, Department of Biology, University of Copenhagen, Copenhagen DK-2100, Denmark; Epigenetics Programme, The Babraham Institute, Babraham, Cambridge CB22 3-AT, UK; Adaptimmune Ltd, Oxfordshire OX14 4RX, UK; Ecology and Evolution, Department of Biology, University of Copenhagen, Copenhagen DK-2100, Denmark; Ecology and Evolution, Department of Biology, University of Copenhagen, Copenhagen DK-2100, Denmark; Department of Molecular Medicine, Aarhus University Hospital, Aarhus DK-8200, Denmark; Epigenetics Programme, The Babraham Institute, Babraham, Cambridge CB22 3-AT, UK; Ecology and Evolution, Department of Biology, University of Copenhagen, Copenhagen DK-2100, Denmark

## Abstract

Circular DNA can arise from all parts of eukaryotic chromosomes. In yeast, circular ribosomal DNA (rDNA) accumulates dramatically as cells age, however little is known about the accumulation of other chromosome-derived circles or the contribution of such circles to genetic variation in aged cells. We profiled circular DNA in *Saccharomyces cerevisiae* populations sampled when young and after extensive aging. Young cells possessed highly diverse circular DNA populations but 94% of the circular DNA were lost after ∼15 divisions, whereas rDNA circles underwent massive accumulation to >95% of circular DNA. Circles present in both young and old cells were characterized by replication origins including circles from unique regions of the genome and repetitive regions: rDNA and telomeric *Y’* regions. We further observed that circles can have flexible inheritance patterns: [*HXT6/7^circle^*] normally segregates to mother cells but in low glucose is present in up to 50% of cells, the majority of which must have inherited this circle from their mother. Interestingly, [*HXT6/7^circle^*] cells are eventually replaced by cells carrying stable chromosomal *HXT6 HXT6/7 HXT7* amplifications, suggesting circular DNAs are intermediates in chromosomal amplifications. In conclusion, the heterogeneity of circular DNA offers flexibility in adaptation, but this heterogeneity is remarkably diminished with age.

## INTRODUCTION

In the unicellular eukaryote *Saccharomyces cerevisiae*, aging is determined by the number of divisions a mother cell undergoes before losing the ability to replicate (1). The asymmetric division of yeast causes an accumulation of factors in the mother cell such as protein aggregates (2,3) and circular DNA from the rDNA locus, also known as ERCs or [*rDNA^circles^*] (4). Accumulation of circular rDNA is associated with a massive increase in ribosomal rRNA synthesis (5) and reduces the number of divisions a cell can undergo by 40% (4). Replicative aging is not specifically associated with circular DNA from the rDNA locus but rather the accumulation of circular DNA in general causes a shorter lifespan of the mother cell (4).

The segregation and accumulation of circular rDNA has been extensively studied (4–10). However, our knowledge about segregation of other circular DNA species of chromosomal origin is based on a few model circles with a small range of genetic markers (4,6,7,11). Therefore, it is not clear whether segregation kinetics, inferred from a small number of model circles, can be generalized to circular DNA formed from other parts of the genome. The segregation kinetics is important to establish since circular DNA derived from other parts of the genome might naturally contribute to aging, and because amplification of genes on circular DNA can have strong phenotypic effects on the host cell (12–16).

Circular DNA from the rDNA array on chromosome XII carries a consensus sequence for replication (autonomously replicating sequence, or ARS) in each rDNA-repeat unit (17). The presence of an ARS likely ensures that an [*rDNA^circle^*] is replicated but does not guarantee faithful segregation between mother and daughter cells in mitosis (6,7,11). The asymmetric segregation of replicated [*rDNA^circles^*] is suggested to be the reason that [*rDNA^circles^*] accumulate in mother cells (4). Circular rDNA was shown to mis-segregate with the mother cell with an estimated retention frequency above 0.99 per circle (10). This segregation bias may explain the accumulation of replicating circular rDNA, although the amount might also be affected by increased *de novo* formation in aging cells (18), available nutrients and polymerase I activity (9), as well as the length of the rDNA array (9).

The fate of circular DNA from other parts of the genome has primarily been studied through plasmids with the marker genes *LYS1* and *URA3* (4,6–8,11). These circular DNAs without centromeres have segregation biases similar to [*rDNA^circles^*] (6,11) and two models have been suggested to explain their tendency to segregate towards the mother cell. In the active retention model, rDNA and non-rDNA circles are actively retained in mother cells by attachment to the nuclear pore complexes via the TREX-2 and SAGA complexes, which are suggested to tether the circular DNA to the nuclear periphery of the mother cell (8). The alternative, known as the diffusion model, suggests that segregation of non-rDNA circles is determined by passive diffusion from the mother nucleus to the daughter nucleus (6). Nuclear geometry restricts diffusion of circular DNA from mother to daughter under mitosis, leading to a segregation bias (6). The passive model is based on *LYS2* circles with and without replication origins that distribute randomly across the nucleus rather than being associated with the nuclear envelope (6). The different circle distributions observed in the nucleus during mitosis suggest that more than one mechanism for segregation bias might exist.

Other genetic elements on circular DNA can ensure more equal segregation between mother and daughter cells. Circles with replication origins and centromeres are mitotically stable through replication and anchoring to the mitotic spindle. Deviation from a 1:1 segregation is expected only when centromeric circles fail to replicate (11). Anchoring of circular DNA to telomeres through telomere-binding proteins also ensures near equal segregation between mother and daughter cells (6,19).

Recent studies show that circular DNA can arise from all parts of eukaryotic genomes (20–23). While the majority of circular DNA is smaller than 1 kilobase (kb) (20,22,24), some circular DNAs are large enough to fully span transposons (25), centromeres, genes, and replication origins (13,20,22,25–27). Hence, extrachromosomal circular DNA can acquire all the genetic elements required for their replication and propagation of their genetic material. Nonetheless, a global overview of how circular DNA segregates and affects aging is still missing.

To obtain a global overview of how circular DNA segregates, we applied the yeast Mother Enrichment Program (MEP) to obtain young and corresponding aged cells (28) and combined MEP with our methods for purification and mapping of circular DNA (29,30). This combined method allowed us to dissect the inheritance pattern of circular DNA while *S. cerevisiae* cells undergo replicative aging. To get a robust measure of the different circular DNA species present in young and aging cells, we developed novel methods for mapping circular DNA from both uniquely mappable and repetitive regions of the *S. cerevisiae* genome. Besides identifying circular DNA with different segregation patterns, we adapted methods for quantifying the copy number levels of the detected circular DNA. This method allowed us to cluster circular DNA species and search for consensus elements that determine their inheritance patterns. We found that most circular DNAs are lost from aging cells, while circular DNA with replication origins are prone to be maintained in cells as they age but only a few of these circular DNAs accumulate in aging cells.

## MATERIALS AND METHODS

### Cells

Strains for preparing circular DNA datasets were: UCC5185 (diploid) with the genotype S288C *MAT***a**/*MAT*α *ade2::hisG*/*ade2::hisGhis3*/*his3leu2*/*leu2LYS2*/*lys2ura3Δ0*/*ura3Δ0trp1Δ63*/*trp1Δ63MET15*/*met15Δ::ADE2hoΔ::PSCW11-cre-EBD78-NATMX*/*hoΔ::PSCW11-creEBD78-NATMXloxP-UBC9-loxP-LEU2*/*loxP-UBC9-loxP-LEU2loxP-CDC20-Intron-loxPHPHMX*/*loxP-CDC20-Intron-loxP-HPHMX* (28). Cells carrying [*GAP1^circle^*] in the CEN.PK background (G1 and G2) served as the positive control for circular DNA (13). Prolonged glucose-limited chemostat cultures were made with CEN.PK wt *MAT*α G6, as in (31).

### Separation of progeny, aged and young populations

MEP 2–5 population number 2–5 (hereafter population 2–5) were grown overnight in YPD medium (2% peptone, 1% yeast extract, 2% glucose) at 30°C with shaking at 200 rpm to mid-log prior to labelling. MEP 6–10 samples for datasets 6–10 were transformed with an empty plasmid (pRS316) for quantification purposes, then individual colonies were picked and grown overnight in synthetic complete medium minus uracil to mid-log phase. Media components were purchased from Formedium and medium was sterilized by filtration. [*GAP1^circle^*] strains (13) were propagated in minimal medium with l-glutamine as the sole nitrogen source to sustain the [*GAP1^circle^*] (2% dextrose, 0.16% yeast nitrogen base without ammonium sulfate and amino acids, 0.34 mM l-glutamine). Upon initiation of experiments, cells were transferred to nonselective conditions, YPD, for 5 generations.

For biotin labelling, 1 × 10^7^ cells were harvested by centrifugation (15 s at 13 000 g), washed twice with 500 μl PBS and re-suspended in 500 μl of PBS containing ∼3 mg/ml Biotin-NHS (Sigma B1022). Cells were incubated for 30 min on a rotating wheel at room temperature, washed once with 125 μl PBS and re-suspended in YPD. One sample of 0.25 × 10^7^ cells was frozen in liquid nitrogen (N_2_) at this point (hereafter young population). For populations 2–5, 0.25 × 10^7^ cells were inoculated in 12 ml YPD in a 25 ml flask and grown for 8 hours at 30°C with shaking before harvest after 5 doublings by centrifugation and freezing in N_2_ (progeny population). Then, 0.5 × 10^7^ cells were inoculated in 500 ml YPD in a 1 l flask containing 1 mM β-estradiol in a 500 ml flask and grown for 48 hours as above before harvest (hereafter referred as aged subpopulation). For datasets 6–10, 0.5 × 10^7^ cells were inoculated in 50 ml YPD in a 100 ml flask and grown for 8 hours at 30°C with shaking, followed by harvesting by centrifugation and freezing in N_2_ (progeny subpopulations). Then, 0.25 × 10^7^ cells were inoculated in 150 ml YPD containing 1 mM β-estradiol in a 500 ml flask and grown for 24 h as above before harvest (aged population).

For cell purification, gradients (1 for progeny population or 2 for aged population) were formed by vortexing 1.16 ml Percoll (Sigma P1644) with 42 μl 5 M NaCl and 98 μl water in 2 ml tubes and centrifuging 15 min at 15 000 g, 4°C. Cells were defrosted and washed 1× with 1 volume of cold PBS + 2 mM EDTA (PBSE) before resuspension in ∼250 μl cold PBSE per gradient and layering on top of the pre-formed gradients. Gradients were centrifuged for 20 min at 1000 g, and the upper phase and brown layer of cell debris were removed and discarded. One ml PBSE was added, mixed by inversion and centrifuged 1 min at 2000 g to pellet the cells, which were re-suspended in 1 ml PBSE per time point (recombining samples split across two gradients). 50 μl streptavidin microbeads were added (Miltenyi Biotech 1010007) and cells incubated for 30 min on a rotating wheel at room temperature. One LS column per sample (Miltenyi Biotech 1050236) was equilibrated with cold PBSE in 4°C room. Cells were loaded on columns, allowed to flow through under gravity, washed with 8 ml cold PBSE and eluted with 1 ml PBSE using a plunger. Cells were re-loaded on the same columns after re-equilibration with ∼500 μl PBSE, washed and re-eluted, and this process repeated for a total of three successive purifications. For quality control, 50 μl cells was set aside and the remainder pelleted by centrifugation and frozen in N_2_ (progeny+ and aged population fractions). For the progeny fraction, the initial flow-through of unbound cells from the first column was collected and passed through a second column to deplete remaining biotin-labelled cells to form the progeny fraction. For quality control, 50 μl cells were diluted to 300 μl final volume containing 0.3% Triton X-100, 0.3 μl 1mg/ml streptavidin 647 (ThermoFisher Scientific S11227) and 0.1 μg/ml DAPI in PBS. Cells were stained for 15 min, washed once with PBS + 0.01% Triton-X100 and resuspended in 7 μl VectaShield (Vector Laboratories H-1000).

The MEP procedure caused an immediate decline in half of the yeast population viability because the MEP selects against new born cells and they were 50% of the culture. Furthermore, the remaining cells underwent a decline in viability over time caused by replicative aging. To remove the effect of the immediate drop in viability from our downstream analyses, we evaluated the initial viability of each culture 2 h after addition of estradiol by plating cells and counting colony forming units, and used this as a baseline for calculating the reduction in viability determined by plating an equivalent volume of culture at later time points.

### Cell counting

Two different methods were used to determine the precise number of cells in experiments. For the *counting chambers* method, we counted at least five different chambers per culture. For the *hemocytometer* method, cell samples were pelleted, fixed in 70% ethanol and counted in a hemocytometer after rehydration in PBS. Approximately 1 × 10^6^ cells were pelleted and frozen in N_2_.

### Circular DNA purification and sequencing

Circular DNA was purified from young, aged and progeny subpopulations of 10^6^ cells using the Circle-Seq protocol (20) with minor changes: Cell membranes were disrupted by 10 units zymolyase (USBiological) for 1.5 h at 35°C. Zymolyase was inactivated at 60°C for 5 min. To ensure sufficient circular DNA precipitation after column chromatography, samples were incubated at –20°C for 45 min and centrifuged at 9788 g for 30 min at 2°C, followed by 70% ice-cold ethanol wash and recentrifugation at 9788 g for 5 min at 2°C. Air-dried DNA was dissolved in 19 μl of sterile water and treated with SwaI in two cycles (2 × 2 FastDigest units, Thermo Scientific) of 1 h at 37°C (total volume 25 μl**)** with heat-inactivation for 15 min at 65°C. To remove all linear chromosomal DNA, four units of exonuclease (Plasmid-Safe ATP-dependent DNase, Epicentre) was added to the reaction at 37°C (reaction volume 40 μl) with fresh 2.5 units DNase added every 24 h with additional ATP and buffer. After digestion for 6 days, enzymes were heat inactivated for 30 min at 70°C. Liquid (∼47 μl) was evaporated to half volume using a vacuum centrifuge (MAXI-DRY LYO, Heto) and 10 μl of the volume was treated with REPLI-g Mini (Qiagen) for 40 h.

Two different sets of plasmid-spiked mixtures were used as internal controls. We used the following plasmid-to-cell ratios: pUG72 (1:1000), pUC19-yEGFP (1:10000), pSH63 (1:1000), p4339 (1:10000), YGPM25009_18kb (1:100), YGPM3k20_chrV_26kb (1:100), pBR322 (1:100) and pRS416 (endogenously maintained) for the populations 2–5. For the populations 6–10, we used the following ratios: pUG72 (1:50), pUC19-yEGFP, (1:500), pSH63, (1:500), p4339 and pRS316 (1:500) and pRS416 (endogenously maintained). pUG72 and pUC19-yEGFP were added when cells were lysed and plasmids pSH63 and p4339 were added after exonuclease treatment. The phi29-amplified circular DNA was subsequently fragmented by sonication and the output sequenced by Illumina (HiSeq 2500) paired-end sequencing with read lengths of 2 × 141 bp for populations 2–5 and 2 × 75 bp for the populations 6–10. Linear DNA content was analyzed in samples during the exonuclease time course using standard PCR methods and primers: *ACT1* 5′-TGGATTCTGGTATGTTCTAGC-3′ and 5′-GAACGACGTGAGTAACACC-3′ and quantitative PCR (qPCR) primers 5′-TCCGTCTGGATTGGTGGTTCTA-3′ and 5′-TGGACCACTTTCGTCGTATTC-3′. Exonuclease-treated total DNA was amplified by phi29 only when all linear *ACT1* was depleted.

### Quantitative PCR

qPCR was performed on a QuantStudio 7 PCR system (Applied Biosystem) with Power SYBR Green PCR Master Mix (Life Technologies, Carlsbad, CA, USA) using the following program: 1 cycle 95°C, 10 min; 40 cycles of 95°C, 15 s and 60°C for 60 s and finally 1 cycle 95°C, 15 s and 60°C 15 s to assess reaction specificity (melting curve). Primers for quantification of [*rDNA^circles^*] were: 5′-CTTCTTCCCAGTAGCCTCATCC-3′ and 5′-TGAACAGTGAACAGTGGGGAC-3′; [*CUP1-1^circles^*], 5′-CTGGCAAGTAGAAAGGAACACC 3′ and 5′-TCTCCGATACCTGCCTCTATTG-3′: [*GAP1^circles^*], 5′-TGTTCTGTCTTCGTCACCGC-3′; 5′-GGCTCTACGGATTCACTGGC-3′; *ACT1*, 5′-TCCGTCTGGATTGGTGGTTCT-3′ and 5′-TGGACCACTTTCGTCGTATTC-3′. All qPCR reactions were performed in quadruplicate and experiments were done a minimum of twice.

### Genotyping of [*Y’telomeric^circle^*], [*ENA^circle^*], [*CEN1^circle^*] and [*GAP1^circle^*]

PCR was carried out according to standard procedures. The following inverse PCR primers were used for detection of circular DNA from: [*ENA5 ENA2 ENA1^circle^*], 936 5′-ACTTGTGCCTATAAATTCAGCGG-3′ and 937 5′-GTACTTCTACTACAATCCATACAGAAG-3′; [*CEN1-1^circle^*], 917 5′ ACGAAGACTGAACTGGACGG 3′ and 918 5′ ACGAAGACTGAACTGGACGG 3′; and [*GAP1^circle^*], 961 5′AACTTTGGGGATTCGGAGTTC3′ and 962 5′-TTGATTACTTGACCCAGAGCG-3′. The [*Y’telomeric^circle^*] was amplified with primers that were general for telomeric *Y’*-elements: 892 5′-GATACGATACTTTCTGGC-3′ and 893 5′-GATGGTGTTAGACAAGGC-3′ providing a 5.4-kb product, and primer 892 and a TEL4R TEL12R TEL15R-specific primer 895 5′-CCCAACATAGAAAACAGAAGG-3′. Purified PCR products were next Sanger sequenced for assembly of the [*Y’telomeric^circle^*] sequence. Sequencing was done with 893, 895, 808 5′-ACCGACATTAACAAAGAGTCG-3′; 842 5′-CTGGATAGCGATGATGTTCC-3′, 896 5′-CCTTCTGTTTTCTATGTTGGG-3′, 897 5′-AGTTGAGTTTCCGTGTCC-3′, 898 5′-TGGTACCTTCTTCTGATGG-3′, 899 5′-GTAACAAATCGAGACATTTCG-3′, 900 5′-ATGGCGTTACTGATTTATACG-3′, and 901 5′-TGACACAGTGGAACTGATAG-3′. *Y’* is identical at TEL4R, TEL12R and TEL15R and could therefore not be separated by sequencing. These circles contained the entire telomerase-encoding *Y*’-element. PCR reactions were carried out with 0.2 ng of template from phi29-amplified sample or 4 μl of template from 130-h exonuclease-treated sample using standard conditions.

### Read alignment

We demultiplexed the reads allowing zero mismatches in the barcodes and checked their quality with FastQC (32). We used the *S. cerevisiae* reference genome (S288C-R64-2-1) and the plasmids included in the Circle-Seq procedure as the alignment reference. Reads were aligned using BWA-MEM (v0.7.17-r1188) (33) with the –q option, leaving the rest of the parameters on their default settings. Samples with a mapping percentage lower than 80% were realigned reducing the seed (-k: 13). We used SAMtools (v1.9) (34) in all our downstream BAM processing steps (Supplementary Figure S1).

### Circular DNA identification

To identify circular DNA from the alignable regions of the yeast genome, we used Circle-Map (v.0.9) package (30). We first extracted all the reads with signs of variation using the subpackage Circle-Map ReadExtractor. Then, we executed the Realignment subpackage with all the parameters set to default. We found that a subset of the circular DNA intervals were called with very few split reads supporting the detection coordinates and without strong read coverage support. To obtain robust circular DNA identification, we iteratively merged the detected circular DNA intervals that reciprocally overlapped by 50%. We kept those with >4 supporting reads and a sequencing coverage of >90%. We evaluated the effect of the sequencing depth on number of circular DNAs by subsampling (setting SAMtools seed to 2) reads on the BAM files at intervals of 5% of reads, and executing the Circle-Map (v.0.9) package as described above.

To detect circular DNA that arose from repetitive regions of the genome, we identified reads that had a highest alignment score relative to the second-highest alignment score of ≥80% with edit distance <3, defining edit distance as the minimum number of base modifications required to transform the read into a perfect match to the reference genome. For every alignment, Circle-Map reported circular DNA coordinates using the leftmost and rightmost alignment position between the highest scoring alignment and the second-highest alignment. Coordinates with read support lower than 20 were removed. To avoid redundant circular DNA calls, circular DNAs reciprocally overlapping by 80% were merged. Finally, a cut-off threshold was applied, removing all putative circular DNA calls with a coverage <80%.

### Genomic feature annotation

We annotated genomic features on circular DNA using bedtools (v2.27.1). We downloaded the features from the Saccharomyces Genome Database (Genome Version R64-2-1). The following features were annotated: replication origins, gene names (symbols) and open reading frames (ORFs) (location holders), centromeres, introns, solo long terminal repeat (LTR) elements, LTR transposons (Ty), telomeric elements, rRNA genes and tRNA genes. We annotated experimentally validated origins of replication from OriDB database (35) transforming the coordinates to the SacCer3 version using the UCSC liftover tool (36).

### Classification of inheritance patterns

Circular DNAs found in both young, progeny and aged populations were determined by intersecting the chromosomal coordinates. We allowed a maximum distance of ±300 bp in the coordinates of circular DNA from alignable regions and 400 bp for those from repetitive regions. We classified the circular DNA as class I if it was in all the subpopulations in a population; class II (if in young and aged), class III (if in young and progeny) and class IV (if only in young). Circular DNA found only in aged or only in progeny subpopulations were classified as circles that had been formed *de novo*. We assigned class V for *de novo* circles found only in aged and/or progeny subpopulations. We applied two post-processing steps. If a circular DNA fit different classes in different populations, we assigned the circular DNA to the highest order of representation (I > II, III & IV > V). Second, circular DNA found in two classes in the same order of representation was considered ambiguous and removed from further analysis.

To investigate if the low level of circular DNA found in both young and aged cells (class I and II) was a consequence of our conservative filtering of the data, we intersected all class IV circular DNA with circles detected in the aged samples using the same distance criteria as above but without applying any read or coverage filters to the data from aged subpopulations.

### *De novo* mutation rate

We calculated *de novo* mutation rates by dividing the number of *de novo* circular DNAs detected in the 2–5 aged yeast subpopulations by cells recovered in the 4 aged populations multiplied by the number of divisions (∼15) of the aged cells.

### Circular DNA quantification

To quantify circular DNA for principal component analysis (PCA) and clustering, we counted the reads aligning unambiguously (quality ≥20) to every circular DNA across samples, and used R (v3.5.1) (37) and the DESeq2 package (v1.22.2) to normalize the read counts with the median of ratios method. We chose the median of the ratios method as it is designed to account for features that have differential abundance between experimental conditions, which is the case for rDNA circles. Finally, for comparison, read counts were log10 transformed. To obtain a better representation of circular DNA frequency in yeast cells, we removed circles not detected in at least two samples as they had little effect on the underlying data distribution (Supplementary Figure S2). Next, we analyzed the recurrent circles using PCA (with scale and center parameters set to true) and hierarchical clustering using the pheatmap (v1.0.12) package.

We used the Manhattan distance for row and column clustering and the average linkage algorithm for defining the clusters. To calculate log fold-change distributions, we combined the circular DNA count matrices from samples 2–5 and 6–10 and computed log_10_-fold changes between the aged and the young subpopulations for all the circles that were detected. We used Gviz (v1.24.0) (38) to visualize the genome coverage of the selected circular DNA.

To quantify the levels of circular DNA from telomeric *Y’*, rDNA and other loci in populations 6–10, we normalized these three classes of reads to the level of spiked-in plasmids. This was done by counting the number of reads aligned to (i) the plasmids; (ii) the telomeric *Y’* locus; (iii) the rDNA locus (with no mapping quality filters due to its tandem duplication structure) and (iv) other circular DNA (quality ≥ 20). Finally, the number of reads aligned to the rDNA, *Y’* locus and other regions were divided by the sum of all reads aligned to plasmids (pUC19_yEGFP, pSH63, p4339 and pRS316).

### Genomic feature overrepresentation

We tested the overrepresentation of the annotated genomic features (OriDB, centromere, LTR, telomere, LTR retrotransposon and intron) and 80 cis-acting element binding sites (39) in the class I and II, and class III and IV circles. To define if a circular DNA carried a *cis*-acting element, we scanned the sequence of the circular DNA for an exact match of the motif, with the exception of the origins of replication, which were allowed non-exact overlaps due to lack of resolution. Then, for every feature, we applied a two-tailed Fisher's test (40). We independently corrected the *P*-values for the cis-acting elements and the genomic features using the Benjamini-Hochberg method.

### Genotyping of cells in prolonged glutamine-limited chemostat cultures

Cells were isolated from a CEN.PK glucose-limited chemostat (CEN.PK wt *MAT*α G6, as in [30]) at generations 0, 35, 71, 82, 106, 124 and 135. DNA was purified from 96 clones from each time point by growing cells in microtiter plates in 300 μl YPGal (1% yeast extract; 2% peptone, 1% galactose). When grown, cells were washed with 0.9 M sorbitol and 0.1 M EDTA and exposed to Zymolyase (Zymo Research) at 37°C until a majority of cells burst when exposed to hypo-osmotic stress in water. Cells were pelleted and suspended in 125 μl P1 buffer (Qiagen) and mixed with 125 μl P2 buffer (Qiagen) and 175 μl of N3 buffer (Qiagen). Denatured cell debris was pelleted by centrifugation and 200 μl supernatant transferred to 200 μl isopropanol for precipitation of DNA after 30 min, 4000 rpm centrifugation. Pelleted DNA was washed in 70% EtOH and suspended in H_2_O. PCR primers used for genotyping were: 654 5′-TACCGAGGTGAGCCCTGC-3′; 655 5′-TCCGTCAGAGGCTGCTACG-3′; 656 5′ CTGACTTCTTCCCACTTTGC-3′; 657 5′-GACAATGGAGAGCAAATGGG-3′; 749 5′-TCTTCAGTTTGACCAGCAACC-3′; and 751 5′-TCGCTTCTCAACAAGATTTGC-3′. A 7.5-kb PCR product from genomic DNA with 655 and 656 and no PCR product from 654 and 655 indicated wildtype *HXT6 HXT7*; a 2.1-kb PCR product from genomic DNA with 656 and 657 indicated a *HXT6/7* deletion; and a 2.7-kb PCR product with primers 654 and 655 indicated amplification of the *HXT6 HXT7* locus. Finally, [*HXT6/7^circle^*] was detected in samples enriched for circular DNA by BamHI digestion and plasmid-safe ATP DNAse (Epicenter E3101K) treatment of linear DNA (BamHI does not cut the *HXT6 HXT7* locus). Samples contained [*HXT6/7^circle^*] if a 2.1-kb product was obtained from exonuclease-treated DNA with the 656 and 657 primer set and no products were obtained with the chromosomal control with primers 749 and 751. Selected PCR products were sequenced by Sanger sequencing to confirm genotypes. Genotyping was repeated on the independent glucose-limited chemostat culture (CEN.PK wt *MAT*α G7 described in [31]).

### Statistical analysis

We used the Wilcoxon test (R v3.5.1) to determine if medians were different between groups.

## RESULTS

### Genome-wide circular DNA detection in young and aged cells

**Figure 1.**
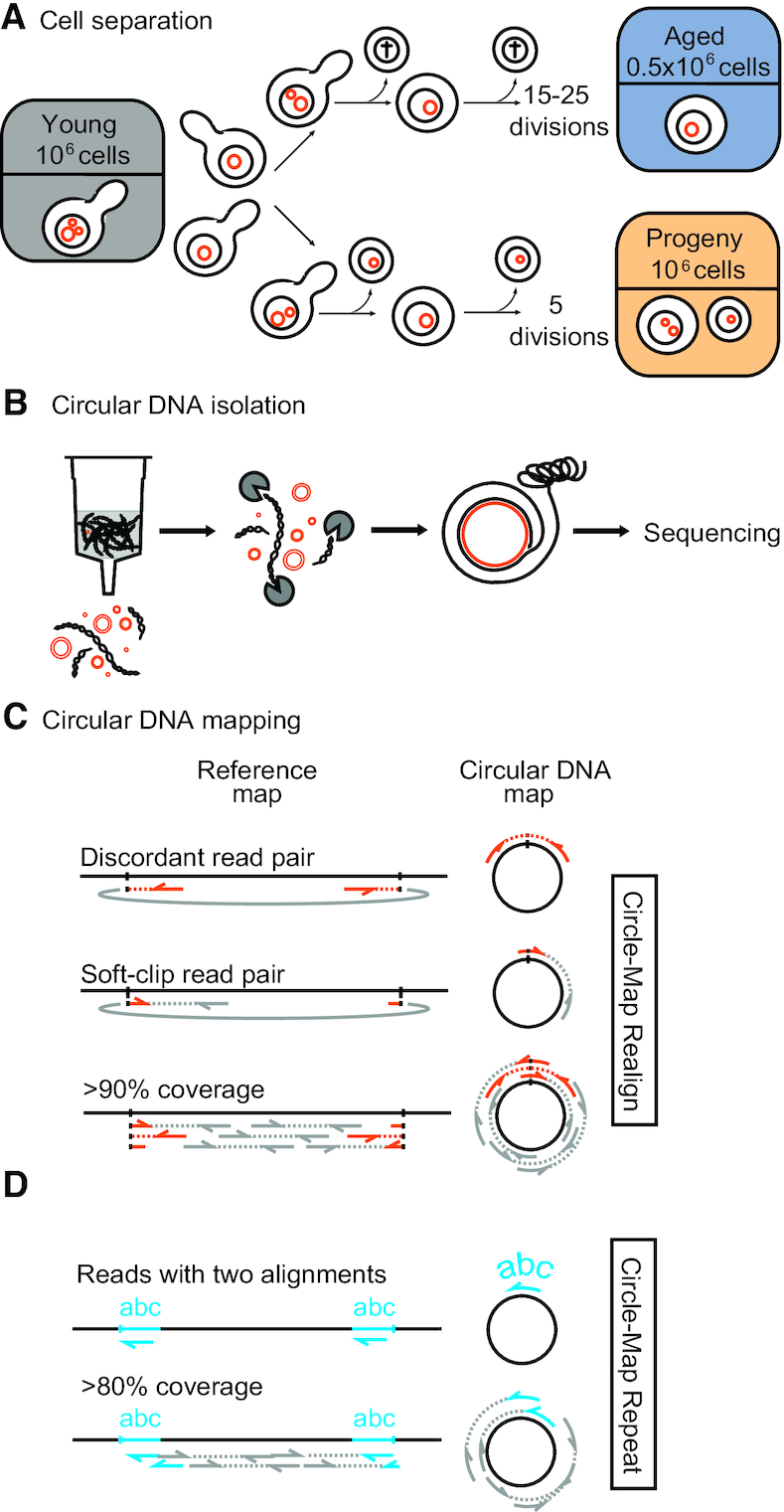
Schematic overview of aged and progeny cell separation using the Mother Enrichment Program, circular DNA purification and mapping. (**A**) Aged, progeny and young cells separated from four populations of yeast cells (2, 3, 4 and 5). The number of cell divisions in aged cells was based on bud-scar counts and the number of cell divisions in the progeny subpopulation was based on a population size that grew from 10^6^ to 32 × 10^6^ when harvested. Only 10^6^ cells were harvested from each of the progeny subpopulations, equally 3.3% of the cells in a population. The aged subpopulations contained on average 7.3 × 10^5^ cells (SD 1.9 × 10^5^). (**B**) Circular DNA purification and sequencing using the Circle-Seq method. (**C**) Circular DNA detection from the mappable parts of the genome using structural variant reads (soft-clipped, discordant and split reads in orange; concordants in gray) and circular DNA read coverage >90%. (**D**) Circular DNA detection from repetitive regions of the genome using reads with a high-scoring suboptimal alignment position (reads with suboptimal alignments in blue; reads without suboptimal alignments in gray) and circular DNA read coverage >80%.

To determine the fate of circular DNA as *S. cerevisiae* cells age, we compared circular DNA across the genome of young and aged yeast populations. This was done in 4 replicate populations, each sampled from a log phase culture primarily consisting of young cells (i.e. 50% of cells never divided before, 25% of cells divided once, which we refer to as ‘young’). The young population was used to produce two separate subpopulations: (i) an ‘aged’ subpopulation of purified mothers that had aged for 48 h (∼15–25 cell divisions) through the MEP (28), which allows only mother cells to divide (Figure 1A); and (ii) a ‘progeny’ subpopulation that was maintained in log phase and allowed to produce progeny for five cell divisions. The progeny population was harvested without further purification (Supplementary Table S1). The viability of MEP cells after 48 h of replicative aging in YPD was determined to be 27% (median = 27.57, *n* = 6, range = 17.14–37.35, Supplementary Table S2), fitting previous reports (28). The remaining inviable cells may or may not have undergone cell death, which could plausibly lead to cell lysis or DNA fragmentation. However, we did not observe enucleated aged cells during quality control of the samples we used, and observed no evidence of DNA fragmentation in Southern blots of equivalently aged and purified MEP cells (41). We therefore consider it highly unlikely that the inviable cells would have undergone DNA loss or fragmentation that could impact our measurements of circular DNA.

We next purified and sequenced circular DNA from each subpopulation by removal of linear DNA (Figure 1B and Supplementary Figure S3), rolling circle amplification and short-read paired-end sequencing as previously described (20). We developed two algorithms to map the origin of circular DNA in the *S. cerevisiae* genome, Circle-Map Realign and Circle-Map Repeats (Figure 1C, D). The first algorithm detects circles from the unique regions of the genome by probabilistic alignment of reads that span the junctions of circles (soft-clipped and discordant reads) (30). Using more than four such junction reads and a read coverage > 90% as cut-off for confident circle detections, we detected 404 unique circular DNAs over the four populations (subpopulation median: 26.5, range: 9–105) (Supplementary Table S3). We found this cut-off to be the optimal number of breakpoint reads and coverage for a robust recording of circular DNA (Supplementary Figure S4). The second algorithm, Circle-Map Repeats, detects circular DNA from repetitive regions of the genome (Figure 1D) such as those from the rDNA array, LTR retrotransposons and the *HXT6 HXT7* locus (4,13,20). We detected 51 repetitive circular DNAs (Supplementary Table S3). Many derived from loci known to undergo circularization such as rDNA, *CUP1 CUP2* and *ENA1 ENA2 ENA5* (4,20,41). In total, we found 455 (51 + 404) circular DNA species using four populations each of young, progeny and aged cells. The experiments were repeated with another four populations replicates of each population where and we found 73 repetitive and 370 unique circular DNAs (Supplementary Figure S5, Supplementary Table S3).

### Young cells have greater genetic heterogeneity from circular DNA than aged cells

**Figure 2.**
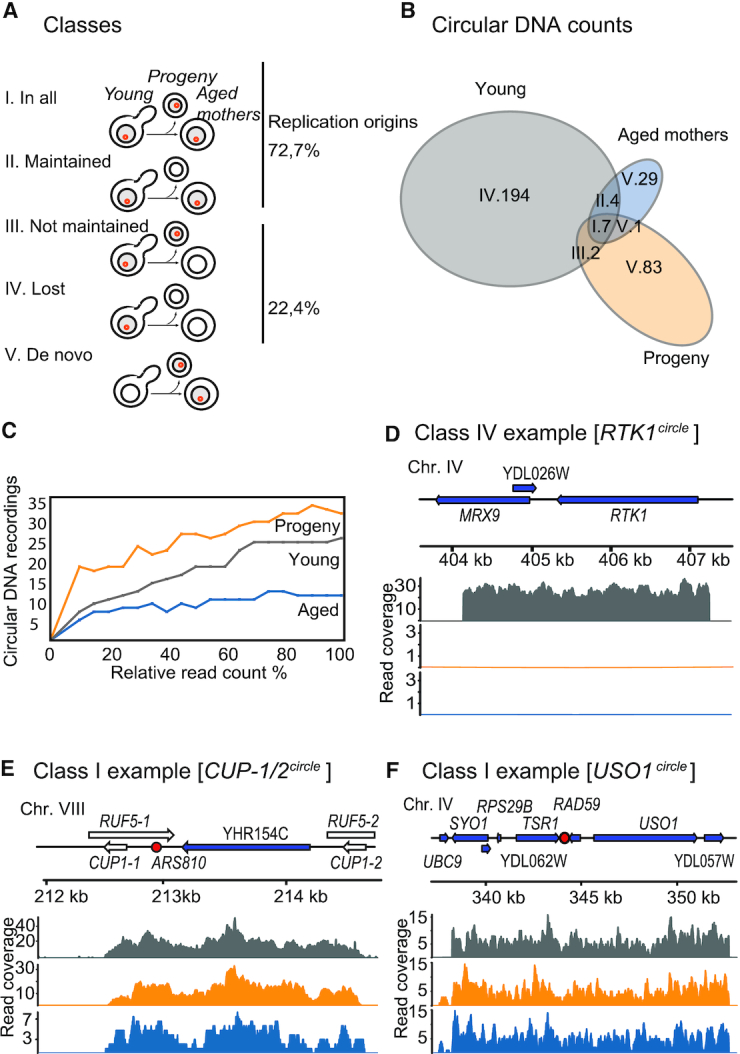
Segregation patterns of circular DNA in yeast cells. (**A**) Schematic overview of different segregation patterns of circles in populations 2, 3, 4 and 5 as cells divide. (**B**) Group-size aware Venn diagram displaying segregation of different DNA circles found in young, progeny and aged yeast subpopulations. (**C**) Saturation plot of circular DNA counts against percentage of reads sampled from the entire alignment file for sample 2. (**D–F**) Circular DNA read coverage plots for young (gray), progeny (orange) and aged (blue) samples. Genomic features present in the circular DNA are shown above coverage plots (repetitive elements in white, genes in blue and replication origins in red). (D) Read coverage plot of [*RTK1^circle^*], a class IV circle. (E, F) Read coverage of [*CUP1/2 ^circle^*] and [*USO1^circle^*], both class I.

**Figure 3.**
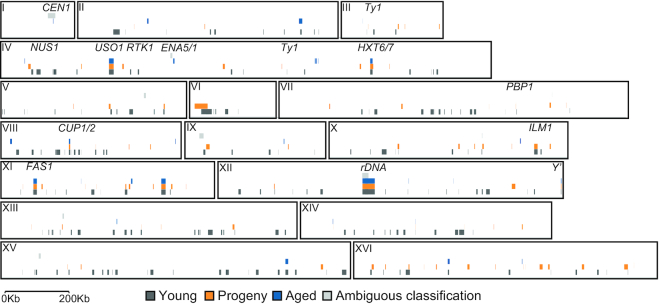
Genomic overview of detected coordinates for all DNA circles found in populations 2, 3, 4 and 5. Each box represents a chromosome with coordinates on the x-axis. Coordinates of circles are in colored lines. Circles that exist in several populations but do not follow the same segregation pattern in all (i.e. ambiguous) are in light gray. Circles in the young subpopulations are in dark gray. Circles in the progeny subpopulations are in orange and circles in aged subpopulations are in blue. Loci used in subsequent analysis are highlighted and shown above.

We next classified the circles found in the young cells into five classes (Figure 2A): (I) circles found in all populations, (II) circles found in both young and aged populations, (III) circles found in the young and progeny but not in the aged, (IV) circles lost from the population and (V) circles formed *de novo*. Our classification revealed that 93.7% of the circles in the young cells (Figure 2B, class IV) were not detected when cells aged, suggesting that the majority of the circles present in young cells were lost as cells aged. The observed circle loss in the aged population could be due to a lack of comprehensive sequencing of our DNA libraries. Hence, we decided to evaluate the effect of sequenced read counts on the circular DNA recordings. We determined this by subsampling a fraction of the total alignments, mapping the circle coordinates and counting the number of detected circles at varying sequencing depths. We reasoned that if the number of detected circles approached saturation as the number of reads increased, we could confirm that the major fraction of the circles present in the sample had already been detected. Using this approach, we found that nearly all circular DNA species were captured at 20–80% of the total reads present in our sequencing datasets (Figure 2C and Supplementary Figure S6A–H). Furthermore, to exclude the possibility that circles in the aged populations were filtered out by our cut-off thresholds, we reanalyzed the overlap between the young and the aged populations without applying split-read and read coverage filters (Figure 1C, D) to the aged samples. This analysis gave no read evidence for circular DNA in aged cells for 96.5% of the class IV circular DNAs found in the young cells (Figure 2D and Supplementary Figure S7A). Hence, our data showed that we detected the majority of circular DNA present in the yeast populations, and that a greater sequencing depth would most likely not have led to detection of more circles in any of the young, progeny or aged subpopulations. In addition to class IV circles that were found only in young cells (Figure 2D), we also found circular DNA that was only in the progeny and/or in aged cells. These circles are likely to be products of *de novo* circularization events (Figure 2A, class V, aged mothers) that occurred during the 15–25 divisions the cells underwent after being separated from the young subpopulation, allowing us to estimate the median DNA circle mutation rate as 7.75 × 10^−6^ mutations/cell division (range: 1.36 × 10^−6^ to 1.17 × 10^−5^). In total, we determined the segregation behavior of 206 circles detected in the young populations, 93 circles in progeny and 42 circles found in the aged populations (Figure 3). We could not determine the segregation behavior of 12 circles as they had different segregation patterns in different samples. We defined these circles as ambiguous and removed them from our downstream computational analyses (Figure 3, gray track). Taken together, these results suggested that the majority of DNA circles present in young cell populations were lost as cells underwent replicative aging for ∼15 divisions.

### Circles present in both young and aged cells are characterized by replication origins

To find traits that could explain why some circular DNAs persist in a population and others do not, we screened for genetic features on circles maintained in the yeast populations as cells underwent replicative aging. We found that the recurring class I and II circles originated from repeat-rich regions of the genome such as the *HXT6 HXT7*, rDNA, and *CUP1-2* loci (Figure 2E, Supplementary Table S3). However, 6 of 11 circles found in this class were from non-repetitive genomic regions (Figure 2F, Supplementary Table S3), and our data indicated no significant enrichment for circles from repetitive regions in the groups formed by class I and class II circles (two-sided Fisher's exact test, *n* = 207, with *P =* 0.09, adjusted for multiple testing). One significantly enriched genomic feature in the class I and II circles was replication origins, found in 8 of the 11 recurring circular DNAs but only in 44 of 196 class III+IV circles (Figure 2A; two-sided Fisher's exact test, *n* = 207, adjusted *P =* 0.006). We found enrichment of telomeric sequences in the class I & II circles, but this result should be handled with care due to the small sample size on this test and the high levels of telomeric circles (4) (two-sided Fisher's exact test, *n* = 207, *P*-v*alue* adjusted for multiple testing *=* 0.0263). We also tested if size or other genomic sequences explained maintenance of circles in cells as they aged. However, neither size of circular DNA nor 80 different transcription factor binding sites (Supplementary Table S4) or sets of genomic features such as LTRs and centromeres (Supplementary Table S5) were significantly overrepresented in the group formed by class I and II circles (two sided Mann–Whitney *U* tests, *n* = 207. *P*-values > 0.05). Taken together, these results suggested that replication origins are an important feature for maintenance of circular DNA as mother cells divide, as recently shown using *CUP1* as a model (41).

### Recurrence of circular DNA from direct repeat loci and loss of a CEN circle

**Figure 4.**
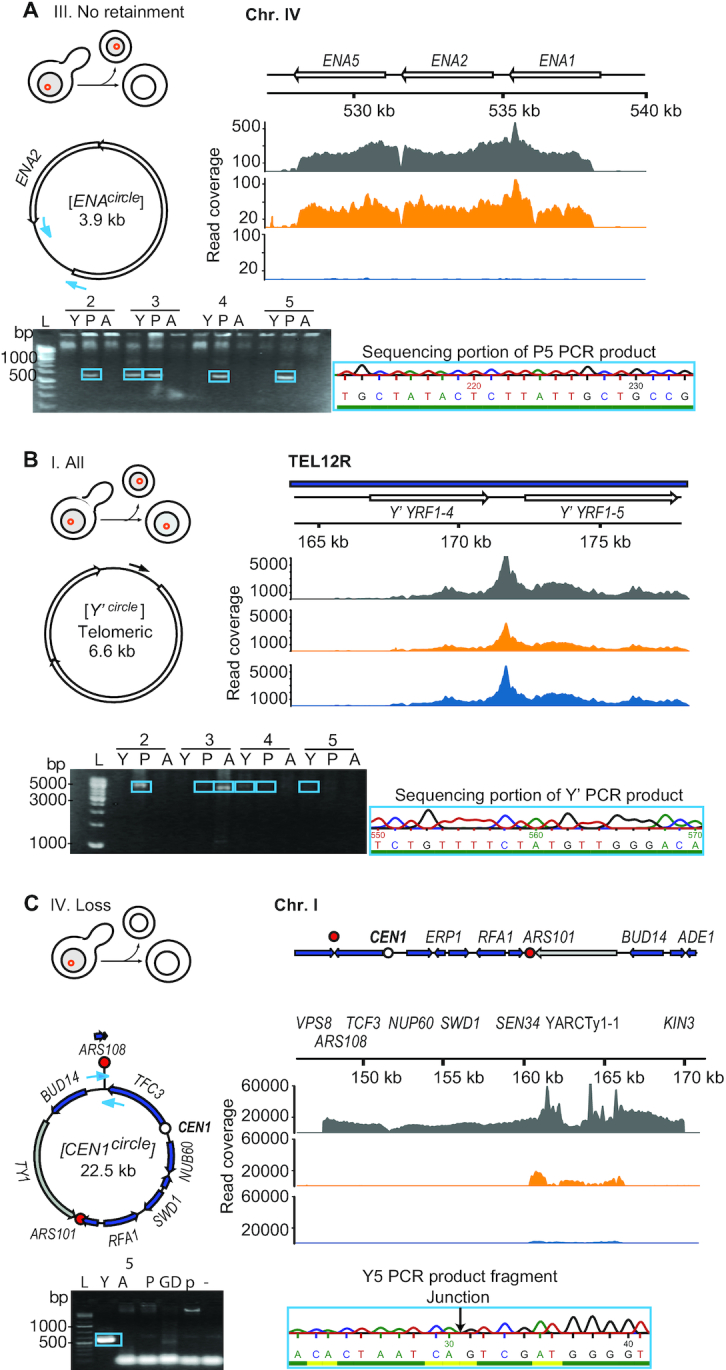
Schematic representation of three validated circular DNAs with their genomic features. Unique genes, blue arrows; repetitive elements, white arrows; retrotransposons, gray arrows; replication origins, red circles; centromeres, white circle. Thin arrows indicate PCR and sequencing primers with the read coverage for different subpopulations (gray, young; orange, progeny; blue, aged). PCR-based gel validation of purified circular DNA (from young subpopulation, Y; progeny subpopulation, P; aged subpopulation, A. Whole genomic DNA, GD; the negative pUG72 plasmid control, p; L, ladder. PCR products were sequenced and part of the Sanger sequencing trace is shown, confirming the identity of a circle from the *ENA* locus (**A**), [*Y’ telomeric ^circle^*] (**B**), and a circular DNA spanning the centromere of chromosome 1 (**C**).

Among the circular DNAs we identified, we found some did not follow the inheritance patterns expected from their genetic features. These were circles from direct repeats, telomeric *Y*’-circles and centromeric circles (Figure 4). An example of a circular DNA from a direct repeat locus was the [*ENA^circle^*], which did not carry a replication origin but still recurred in different subpopulations (Figure 4A, circular DNA diagram). Consequently, we expected [*ENA^circles^*] would fail to be maintained in the population as they lack replicative capacity. [*ENA^circle^*] forms by recombination (20) between the paralogous *ENA* genes on chromosome IV. We detected [*ENA^circles^*] in the young and progeny subpopulations, yet did not detect the circles in any of the corresponding aged subpopulations (Figure 4A, genome coverage plot, PCR and Sanger sequencing), suggesting that [*ENA^circles^*] formed with a high rate and were lost from the population after fewer than 15–25 cell cycles.

Another class of recurring DNA circles came from *Y’* telomeric elements (42). Due to the poor alignment of telomeric regions, we could not determine the genomic origin of the [*Y’ telomeric^circle^*] based on short-read sequencing. We therefore used a combination of PCR and Sanger sequencing on purified circular DNA to determine the genomic origin of the [*Y’ telomeric^circle^*] in aged subpopulations. Sanger sequencing revealed that [*Y’ telomeric^circle^*] could derive from three telomeric (TEL) loci, the right arm of chromosome IV or XV that each carry one copy of the sequence or the right arm of chromosome XII, TEL12R, which carries tandem directed *Y’* repeats. The [*Y’ telomeric^circle^*] sequence matched exactly the middle of the two repeats on TEL12R (Figure 4B, Supplementary File S1). Thus, we anticipate that the [*Y’ telomeric ^circle^*] primarily formed from TEL12R because this circle was present in several populations. These traits also suggest high formation rates, likely because of more frequent events between the Y’-elements in direct repeats on TEL12R than non-homologous ends of *Y’* from other chromosomes. Finally, telomeric *Y’* circles did not carry X core-repeats, as expected if they were derived from TEL4R or TEL15R. Taken together, these results suggested that the homologous direct repeat loci TEL12R and *ENA1 ENA2 ENA5* formed circular DNA at high rates.

Circular DNA spanning centromeres are intriguing, as they contain the required genomic signal to segregate faithfully in mitosis and are potential intermediates in the evolution of new chromosomes with altered synteny. We scanned the detected circular DNA (Supplementary Table S3) for the presence of intact centromeres and discovered a 22.5-kb large circular DNA that contained the centromere of chromosome I, two replication origins, a LTR retrotransposon (Ty1 element) and 7 genes (Figure 4C). Hence, this circular DNA carried all the necessary elements for replication and equal segregation. Nonetheless, we found the CEN circle in one young subpopulation and not in the corresponding aged or progeny populations (Figure 4C). This indicated that the [*CEN^circle^*] was lost from the aged yeast populations despite having a centromere.

### Replicative aging is associated with loss of genetic heterogeneity from circular DNA

**Figure 5.**
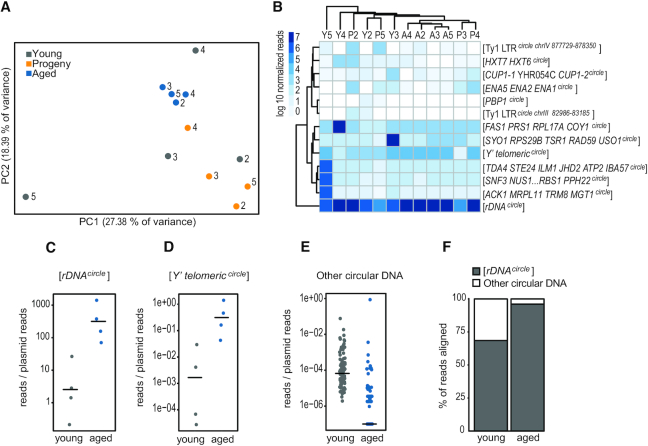
Quantification of circular DNA as cells undergo many divisions. (**A**) Principal component analysis of circular DNA found in at least two samples from populations 2, 3, 4 and 5. Axes, first principal component (PC1) against second principal component (PC2). (**B**) Hierarchical clustering of recurrent circles in (A). Clustering of similar samples is in the column dendrogram and clustering of similar circles is in the row dendrogram. Read coverage scale is in the upper left corner. (C–E) Relative levels of [*rDNA^circle^*] (**C**), [*Y’ telomeric ^circle^*] (**D**) and DNA circles from other genomic loci (**E**), calculated by normalizing read counts to spiked-in plasmids in populations 6–10. Horizontal black lines indicate median value. (**F**) Percentage of circular DNA sequence reads aligning to the rDNA locus (gray) and to other genomic loci (white) in young and aged subpopulations of populations 2–5.

One of the hallmarks of aged yeast cells is the massive accumulation of [*rDNA^circles^*] compared to their young counterparts (4,18,43). To investigate if recurrent non-rDNA circles also accumulated in aging cells, we quantified circular DNA by counting the number of reads aligned to the different circular DNA coordinates and normalizing the read counts using the median of ratios method (44). We chose the median of the ratios method as it is robust and accounts for differentially abundant circular DNA species with high read counts (i.e. [*rDNA^circles^*]) that would otherwise affect the read-count distribution of data. We then removed circles that appeared in only one yeast subpopulation, leading to a set of 13 recurrent circular DNAs (Supplementary Table S6). PCA revealed that the aged yeast populations clustered tightly together (Figure 5A). Young and progeny subpopulations did not form any apparent clusters in the PCA, supporting that aging subpopulations were less heterogeneous than young subpopulations. To obtain a better understanding of why circular DNA profiles from old yeast cells were more similar to each other than to circular DNA profiles from young or progeny populations, we complemented our PCA analysis with hierarchical clustering of the recurrent circular DNA (Figure 5B). As in the PCA results, the sample dendrogram of the hierarchical clustering (Figure 5B, top) had data from the aged subpopulations grouped together, while the young and progeny populations did not form an apparent cluster. When we examined the cells of the hierarchical clustering individually, it became clear that the signal for the aged populations represented mainly [*rDNA^circles^*] and [*Y’ telomeric^circles^*] (Figure 5B). The remaining recurrent circular DNA did not show any evidence of accumulation in aged populations for circles with or without replication origins. To confirm our results, we performed PCA and hierarchical clustering analyses on another four independent yeast populations of young, aged and progeny cells (Supplementary Figure S8A, B).

To more precisely determine accumulation of [*rDNA^circles^*] and [*Y’ telomeric^circles^*] in the aged samples, we purified and sequenced circular DNA in the presences of transformed and spiked-in plasmids in four populations, samples 6–10. This method allowed us to normalize read count values between the young and aged subpopulations (Figure 5C–E, Supplementary Tables S7-S9) for semi-quantitative measuring of circular DNAs between samples. [*rDNA^circles^*] (Figure 5C, Supplementary Table S7) accumulated significantly in the aged samples (median fold-change 432.3, Wilcoxon two-sided test, *n* = 8, *P* = 0.028). Similarly, we found a significant accumulation of [*Y’ telomeric^circles^*] (Figure 5D, Supplementary Table S8, median fold-change 6427.58, Wilcoxon two-sided test, *n* = 8, *P =* 0.028), while circular DNA from other parts of the genome did not accumulate significantly (Figure 5E, Supplementary Table S9). We next addressed how rDNA levels influenced the circular DNA read distribution in the yeast 2–5 populations (Figure 5F). Young yeast populations were already dominated by reads coming from the rDNA locus (median percentage of aligned reads 67.57%, range: 47.78–93.23%). Nonetheless, the percentage increased drastically for the aged yeast populations (median 98.55%, range: 87.37–99.53%), in which reads originating from [*rDNA^circles^*] dominated the samples over circular DNA formed from other genomic loci. We found a similar [*rDNA^circles^*] accumulation pattern in another four yeast populations (Supplementary Figure S8C). Finally, we confirmed the accumulation of [*rDNA^circles^*] using the 2–5 aged populations and qPCR (Supplementary Figure S9).

To confirm that DNA circles from other parts of the genome did not accumulate in aged cells, we measured the level of purified circular DNA upon exonuclease treatment with qPCR. Quantification of two circles that did not accumulate in populations 2–5, [*CUP1/2^circle^*] and [*GAP1^circle^*], from two independent MEP-enriched yeast populations, confirmed that [*CUP1/2^circle^*] and [*GAP1^circle^*] levels were either unaltered or decreased in aged subpopulations compared to young subpopulations (Supplementary Figure S10A–C). Taken together, these results showed that aging cells primarily accumulated [*rDNA^circle^*] and that the number and level of non-rDNA circles generally decreased as cells aged.

### [*HXT6/7^circle^*] alters inheritance patterns when selected for and is detected before stable chromosomal *HXT6/7* amplifications

**Figure 6.**
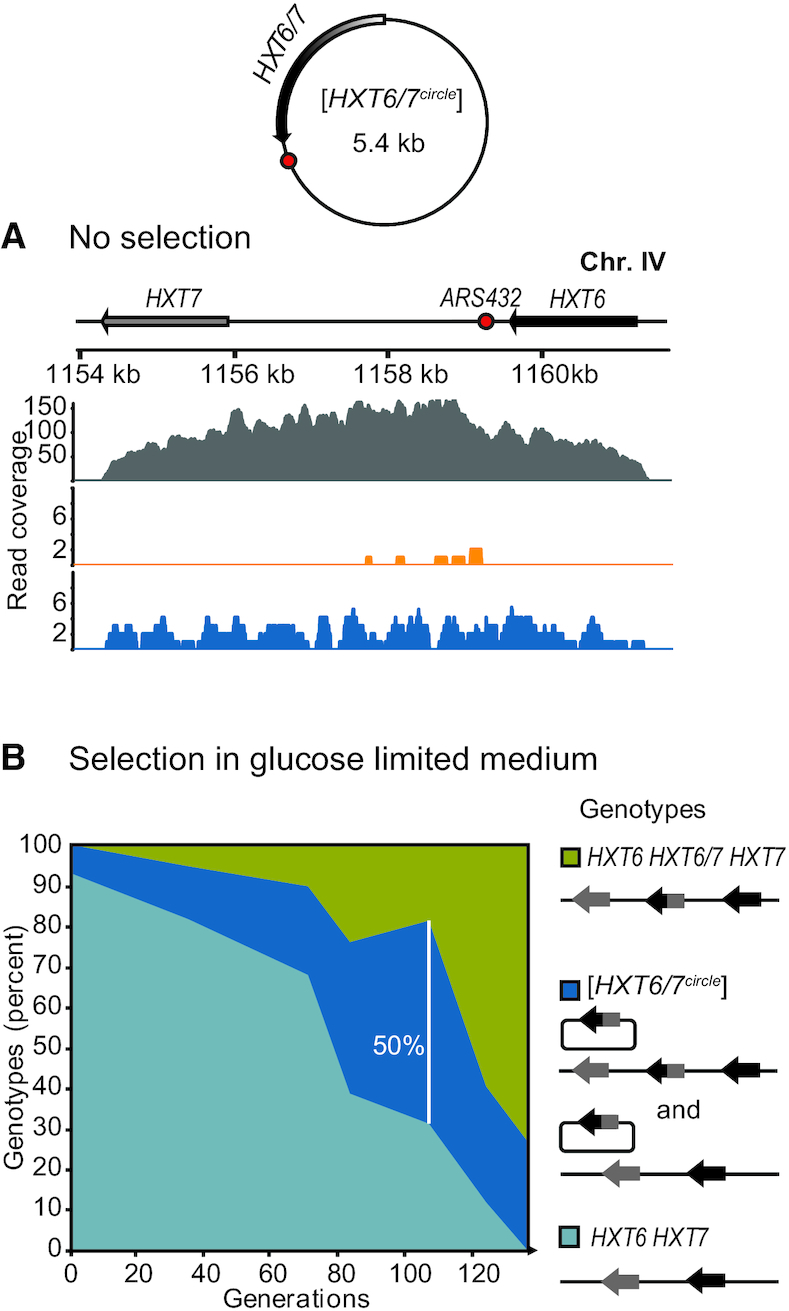
Segregation behavior of circular DNA formed from the *HXT6/7* locus under non-selective and selective pressure. (**A**) Read coverage plot of [*HXT6/7^circle^*] under no selection (detected in subpopulations of population 4). Read coverage is shown for young (gray), progeny (orange) and aged (blue) subpopulations. Replication origin is a red circle in schematic diagrams. Paralogous genes *HXT6* and *HXT7* are in black and gray, respectively. (**B**) *HXT6 HXT7* genotypes in a population of cells exposed to prolonged glucose limitation for 135 generations in a glucose-limited chemostat (genotyped at generations: 0, 35, 71, 82, 106, 124 and 135). Clones with wild-type genotype, light blue; circular amplification, dark blue; chromosomal amplification, green).

Circular DNAs are enriched under conditions that select for them (13,45). We hypothesized that different selective regimes would change inheritance patterns of circular DNA from failure to be maintained (class III and IV) towards occurrence in all cell types (class I), or *vice versa*. To study the inheritance pattern of a circular DNA under selective pressure, we followed the distribution of [*HXT6/7^circle^*] in a population of cells exposed to prolonged glucose limitation. The *HXT6* and *HXT7* genes encode high-affinity glucose transporters, and growth under glucose limitation selects for chromosomal amplification of the *HXT* genes at the *HXT6 HXT7* locus to create a *HXT6 HXT6/7 HXT7* locus (31,46,47). The *HXT* locus can also excise as a [*HXT6/7^circle^*] that contains the 5′-end of *HXT6* and the 3′-end of *HXT7* (Figure 6A, Supplementary Table S3) (20,29). However, the [*HXT6/7^circle^*] has not yet been associated with amplification of the *HXT6 HXT7* locus.

[*HXT6/7^circle^*] was discovered in four aged subpopulations, but only once in the progeny population, suggesting that [*HXT6/7^circle^*] had a segregation bias towards mother cells, similar to other class II circles (Figure 6A). We next investigated the inheritance of [*HXT6/7^circle^*] in daughter cells under conditions that select for retention of [*HXT6/7^circle^*]. In glucose limitation, cells with [*HXT6/7^circle^*] outcompete cells that lack it. We tracked the *HXT6 HXT7* genotypes of individual cells over 135 generations of glucose limitation (Figure 6B), taking samples at time points of 0, 35, 71, 82, 106, 124 and 135 generations. Initially, the majority of the genotypes were wild-type (93.02%), with a minor fraction of cells carrying the [*HXT6/7^circle^*] (6.98%) and no stable chromosomal amplifications. As selection progressed, the genotypes switched towards cells carrying [*HXT6/7^circle^*] and eventually shifted to cells with chromosomal *HXT6 HXT6/7 HXT7* amplifications dominating the population. The fact that a large number of cells (50% of cells at generation 106) contained the [*HXT6/7^circle^*] suggested that [*HXT6/7^circle^*] was inherited by daughter cells under selective pressure. Exponentially growing cells consist of 50% young daughter cells that have never divided, 25% that have been mothers once, and 12.5% that have been mothers twice, with additional populations according to this pattern. Therefore, a large proportion of the cells carrying [*HXT6/7^circle^*] under glucose-limited conditions must be daughters that received [*HXT6/7^circle^*] from their mother. In short, inheritance patterns of circular DNA shifted under different selective regimes. Furthermore, the progressive accumulation of the [*HXT6/7^circle^*] phenotype followed by stabilization of the amplification genotype suggested that circular DNAs serve as intermediate units for the formation of stable chromosomal amplifications.

## DISCUSSION

Our results suggest that circular DNA of chromosomal origin can follow three different inheritance patterns in cells as they undergo replication. A majority of circular DNA is lost and only a small fraction of all circular DNA appears to be maintained as cells replicate. A subgroup of maintained circular DNAs are [*rDNA^c^^ircles^*] that accumulate at higher levels than other circular species as cells age. This eventually results in aged cells with relatively low genetic variation in circular DNA and a high accumulation of [*rDNA^circles^*].

Circles found in both young and aged cells were characterized by replication origins, suggesting that replication is essential for maintenance of circular DNA. This finding supports previous studies that used acentric plasmids containing replication origins as a model to study the behavior of circular DNA during cell division (8,11). When cells containing these plasmids were grown on non-selective medium, the segregation bias was towards the mother cell, leaving the daughter cell with few or no copies of the plasmid (8,11). The presence of an origin of replication does not solely define the maintenance of a circle, as we found many circles with replication origins were lost. Other factors such as length of mitosis (6) are likely to also contribute to the fate of a circular DNA during cell division.

Our results suggest that the majority of replicating circles do not accumulate to the same extent as [*rDNA^circles^*] and [*Y’* telomeric*^circles^*] in aging cells. We propose that the discrepancy between [*rDNA^circles^*] and other circles is caused either by an accumulation mechanism that is specific for [*rDNA^circles^*] or a very high formation rate from the rDNA locus. Barral and co-workers suggested active retention of [*rDNA^circles^*] and other circular DNA in mother cells occurs through the binding of circular DNA to the SAGA complex and nuclear pore complexes (8). This model does not distinguish between [*rDNA^circles^*] and other circular DNA with replication origins and can therefore not explain the discrepancy in their levels. Circular rDNA formation increases as cells age (5,18) and this could contribute to their accumulation. The rDNA locus is sequestered in the nucleolus in a different enzymatic environment than other parts of the nucleus, with other DNA polymerases that can also influence formation rate (9). The physical separation of nucleus and nucleolus makes separate segregation mechanisms for [*rDNA^circles^*] and other circular DNAs feasible, as recently suggested (5). Recent studies indicate that the level of [rDNA*^circles^*] also depends on the length of the chromosomal rDNA repeat (9). We found that [rDNA*^circles^*] are already present in young cell populations, presumably carried by a minor fraction of aged cells. Then, as the cells age, [rDNA*^circles^*] become the dominant circular DNA form in the population. The accumulation of [rDNA*^circles^*] in aging cells over other circular DNA might be partly a consequence of rDNA copy-number regulation. However, how the self-limiting production of [rDNA*^circles^*] interacts with their accumulation in aging cells is so far unknown. Hence, we currently cannot distinguish if the high level of [rDNA*^circles^*] compared to other maintained circles (Figure 5F) is caused by different segregation mechanisms (passive or active retention) or because circular DNAs have different formation rates but the same segregation mechanism.

An important exception to the inheritance patterns of most non-rDNA circles is circles from genes in direct repeats (*ENA1 ENA2 ENA5, Y’* telomeric and *HXT6 HXT7*). Apparently, *ENA1 ENA2 ENA5* does not have an active replication origin so its recurrence in independent populations (Figure 4) suggests that it is formed *de novo* at high rates. *CUP1 CUP2, Y’* and *HXT6 HXT7* carry replication origins, and are also found recurrently, suggesting that in general, circles from direct repeats have a high formation rate. Hence, our data supports the suggestion from previous work (4,41) that at least three factors determine how circular DNA is enriched and maintained in a cell lineage: the rate at which it is formed from its chromosomal locus, its ability to replicate, and its mode of segregation.

Interestingly, we found that aged cells have a fewer different circular DNAs than young cells. This suggests that yeast cells lose circular DNA genetic heterogeneity through aging and that the genetic variation upon which selection can act becomes lower with age. Young cells have a larger pool of genetic variation than old cells, theoretically allowing young cells to adapt and respond a wider set of conditions than old cells. Furthermore, genes linked to replication origins and direct repeats are more likely to reside on circular DNA and evade the 1:1 segregation pattern, thereby contributing to genetic variation in the form of circular DNA. This finding implies that replication origin-linked genes might evolve faster, as they exist longer on circular DNA in a given population when not under selective pressure. Several genes in direct repeats are known to evolve rapidly in yeast populations (48) and circular DNA could be an intermediate between deletion and amplification mutations. While we cannot rule out that we did not detect a minor fraction of the circular DNA in the aged populations, our computational experiments suggest that we already found the majority of circular DNAs present in the young, progeny and aged populations at 20% sequencing depth. Hence, we are confident that our circular DNA loss rate is not overestimated.

An example of a circle with high formation rates and that has a replication origin is [*HXT6/7^circle^*], which carries a hybrid of two high-affinity hexose transporter genes from the *HXT6 HXT7* locus. We found this circle recurrently in our previous whole genome circles studies (20,29) and it appears repeatedly in independent populations. Results from others show that glucose limitation selects for amplification of the *HXT6 HXT7* locus to *HXT6 HXT6*/*7 HXT7* (46,47). Our data suggest that cells with [*HXT6/7^circle^*] are selected for under glucose limitation, and the proportion of cells with [*HXT6/7^circle^*] increases in the population until cells with stable chromosomal amplifications arise and take over. We propose that [*HXT6/7^circle^*] can be an intermediate in the formation of the more stable *HXT6 HXT6/7 HXT7* chromosomal amplification, and that circular DNAs can facilitate a fast adaptation to selective conditions via a high formation rate, replication and copy number variation.

Amplification of genes in direct repeats through a circular intermediate might occur with other genes in direct repeats in yeast and other eukaryotes such as humans. In humans, genes in direct repeats are known to amplify to lead to disorders such as Charcot-Marie-Tooth disease or Williams-Beuren syndrome (49). In yeast, the [*Y’* telomeric*^circle^*] is suggested to be the intermediate in telomeric extensions in type I survivors of telomerase-deficient cells (42). The origin of [*Y’* telomeric*^circles^*] is unknown but our study suggests they arise primarily from the *Y’*-repeat in TEL12R, possibly due to its structure with two tandem *Y'* repeats.

Class IV circles were detected only in young cells. Most might not have replicated due to a lack of an origin, so they existed only in one copy that we recorded in a young population. Alternatively, class IV circles might have replicated rarely and diffused to daughter cells. Because we recorded circular DNA from only 3% of progeny populations, our analysis would have missed these circles in the progeny. We also found that a [*CEN1^circle^*] was lost during replicative aging. While this circle was expected to segregate 1:1, cells carrying it might have been lost from the aged population if it conferred a decrease in fitness. Cells with a [*CEN1^circle^*] are expected to have lost major fragments of chromosome I, which is expected to result in much lower fitness compared to wild type diploids.

**Figure 7.**
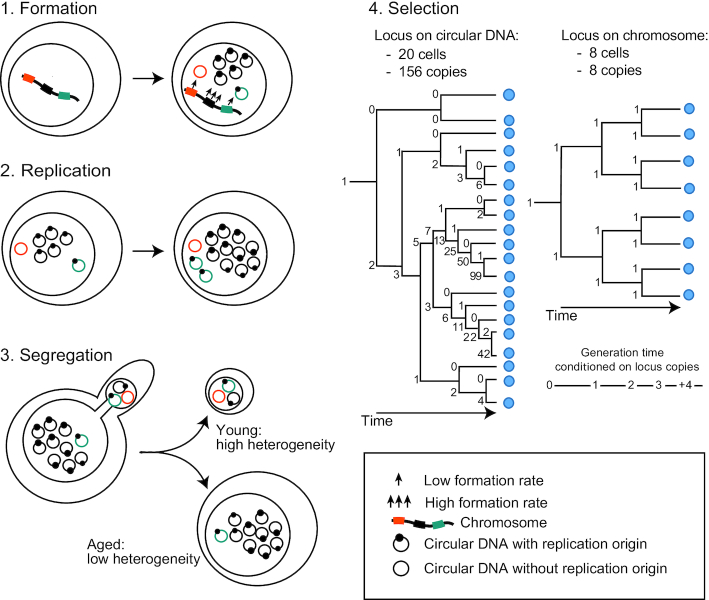
Model figure representing the four factors determining the maintenance of circular DNA in a cell lineage. The formation rate (1): loci with high formation rate (3 up-facing arrows) give rise to circular DNA more often than loci with low formation rates (1 up-facing arrow). Replication (2): DNA circles that lack replicative potential are rapidly lost through dilution on cell division. Segregation (3): 1:1 segregation of a replicated circular DNA to mother and daughter prevents circular DNAs becoming restricted to a sub-population of cells, and therefore maintains the heterogeneity of circular DNAs across the cell population. In contrast, asymmetric segregation increases the copy number of an individual type of circular DNA. Selection (4): a gene that bestows a growth advantage can increase in copy number more rapidly if present on a circular DNA (left tree) than the same gene in a chromosomal location (right tree) due to asymmetric segregation events followed by positive selection for higher copy number. If generation time (horizontal lines) is inversely proportional to gene copy number (shown by numbers), average copy number can rise dramatically in a short time. Time is represented on the X-axis and cells as blue spheres.

Our results allow us to present a unifying model describing four factors that determine the existence of a circular DNA in a cell lineage (Figure 7): the rate by which it is formed from its chromosomal locus, its ability to replicate, its mode of segregation, and the selective advantage or disadvantage it provides. Our data suggest that rDNA circles are the dominant circles in replicative aged cells and that young cells have the largest variety of different circular DNAs. Different parts of the genome appear to have large differences in their propensity to evolve through circular DNA, since loci linked to direct repeats and replication origins are more likely to reside on circular DNA. Selection can furthermore change the inheritance pattern of a circle and lead to rapid propagation of circle-carrying cells in a population. These observations are relevant not only for yeast but are also important for our understanding of aging in other organisms and our insight into evolution of cancer cells through circularized proto-oncogenes.

## DATA AVAILABILITY

Circle-Map Repeats is open source and available in the GitHub repository (https://github.com/iprada/Circle-Map).

The raw sequence data have been deposited on the Sequence Read Archive and can be accessed with no restrictions using the BioProject ID PRJNA593745.

## Supplementary Material

gkaa545_Supplemental_Files
