# Supplementary material for: Replicative aging is associated with loss of genetic heterogeneity from extrachromosomal circular DNA in Saccharomyces cerevisiae
Source: Nucleic Acids Res. 2020 Jul 1;48(14):7883–98. doi: 10.1093/nar/gkaa545 (PMC7430651; doi:10.1093/nar/gkaa545)

Supplementary Figure legends

Supplementary figure S1. Flowchart describing the bioinformatics workflow conducted to obtain circular DNA coordinate files from the raw data. Parallelograms indicate input/output files. Squares indicate programs used during the workflow. Rhombuses indicate filters applied to the data.

Supplementary figure S2. Principal component analysis of the circular DNA found in 2-5 yeast populations without removing the circles found in 1 population only. The axis represent the first principal component (PC1) against the second principal component (PC2).

Supplementary figure S3. (*A*) Confirmation of linear DNA removal with qPCR on the dosage sensitive gene *ACT1.* The upper panel shows the raw cycle threshold values (CT) for *ACT1* qPCR. (*B*) *ACT1* values normalized to the nuclei count for the B2-5 samples. Young samples are indicated as Y, progeny samples are indicated as P and aged samples are indicated as A. NTC indicates non template control.

Supplementary figure S4. Effect of breakpoint reads and circular DNA sequencing coverage on circular DNA detection. Young populations are indicated with Y, progeny populations are indicated with P, aged populations are indicated with A and aged populations selected with the biotinylation program are indicated with A^+. (*A*) Number of detected circular DNA against the number reads crossing the circular DNA breakpoint (split and discordant reads) in the 2-5 yeast populations. (*B*) Number of detected circular DNA against the sequencing coverage within the detected circular DNA coordinates in the 2-5 yeast populations. (*C*) Number of detected circular DNA against the number of reads crossing the circular DNA breakpoint (split and discordant reads) in the 6-10 populations. (*D*) Number of detected circular DNA against the sequencing coverage within the detected circular DNA coordinates in the 6-10 yeast populations.

Supplementary figure S5. Experimental design and circular DNA segregation patterns for the 6-10 yeast populations. (*A*) Schematic representation of the different circular DNA segregation patterns. (*B*) Schematic overview of young, progeny and aged cell separation from 4 yeast populations using the biotinylation program. (*C*) Venn diagram displaying the different circular DNA segregation patterns found on the 6-10 yeast populations separated with the biotinylation experiment. (*D*) Venn diagram displaying the different circular DNA segregation patterns found in the 6-10 populations separated using the mother enrichment program.

Supplementary figure S6. Effect of sequencing depth on the number of circular DNA detections. (*A-D*) Number of detected circular DNA against the percentage of reads sampled from the alignment file in the 2-5 yeast populations. (*E-H*) Number of detected circular DNA against the percentage of reads sampled from the alignment file in the 6-10 yeast populations.

Supplementary figure S7. *(A)* Number of circular DNA found in the young population containing read evidence in the aged population alignment files. Read evidence was obtained by removing the breakpoint read filter (>4) and coverage filter (>90%) we applied in our circle calling strategy.

Supplementary figure S8. Quantification of circular DNA as cells undergoes many divisions. (*A*) Principal component analysis of the circular DNA found in at least two sub-populations of the 6-10 yeast populations. The axis represent the first principal component (PC1) against the second principal component (PC2). (*B*) Hierarchical clustering of the circular DNA detected in at least two samples of the 6-10 populations. The left dendrogram shows the clustering order of the circles (rows), with circle names indicated on the left. The dendrogram below shows the clustering order of the samples (column), with the

sample names indicated in the top part of the plot (Y: young, P: progeny; A: aged with MEP and A+: aged with biotynilation). The normalized read coverage is shown on the upper left part. (*C*) Relative levels of [*rDNA*^{circle}] and circles form other parts of the genome present in the 6-10 yeast populations.

Supplementary figure S9. Quantification of [*rDNA*^{circle}] levels on the young (Y), progeny (P) and aged (A) subpopulations of the 2-5 samples by performing qPCR on the *NTS1* gene after removal of the linear DNA. The upper panel shows the raw cycle threshold values (CT) and the lower panel shows the *NTS1* values normalized to the cell counts and the dosage sensitive gene *ACT1*. NTC indicates non template control.

Supplementary figure S10. Quantification of the *GAP1* and *CUP1* levels on the young (Y), progeny (P) and aged (A) subpopulations of the G1-G2 after removal of the linear DNA. (*A*) Confirmation of the linear DNA removal with qPCR on the dosage sensitive gene *ACT1*. The upper panel shows the raw cycle threshold values (CT) and the lower panel shows *ACT1* copies normalized to the cell counts. (*B*) Schematic representation of the genomic features present on the [*GAP1*^{circle}] (right part). The black arrows indicate the primer location used for the qPCR. The right part shows the qPCR CT values (upper panel) and the cell count normalized gene copies (lower panel). (*C*) Schematic representation of the genomic features present on the [*CUP1*^{circle}] (right). The black arrows indicate the location of the primer pairs used for qPCR quantification. The right part shows the qPCR CT values (upper panel) and cell count normalized gene copies (lower panel).The white dots in the upper panels show the median value. The # in the labels of the axis shows technical replicates.

Population	young	progeny	aged
2	1.00011E6	1.0003E6	0.64568E6
3	1.000644E6	1.0002E6	0.64515E6
4	0.9996E6	0.999924E6	0.6254E6
5	1.0002E6	1.00011E6	1.03E6

Supplementary Table S1. Tab separated file containing the cell counts indicating the number of cells recovered from every young, progeny and aged population. Ordered from left to right, the columns contain the following information: 1, yeast population; 2, cell counts in the young subpopulation; 3, cell counts in the progeny subpopulation and 4, cell counts in the aged subpopulation.

24h	48h
69.811320	31.132075
61.904761	17.142857
63.736263	29.670329
87.951807	37.349397
62.264150	25.471698
66.363636	20

Supplementary Table S2. Tab separated file containing the viability estimates at 24 and 48 hours of six independent aged yeast populations enriched with the mother enrichment program. The viability estimates are show as % of viable cells.

chrom	start	end	discordan	soft-clipp	meancov	covstd	start_ratic	end_ratio	continuity	sgd_featu	sgd_comp	orf	orf_compl	length	segregatic	pipeline	samples	MEP
chrIV	338268	352701	4	4	3.869951	2.2571	1	1	0.030278	SYO1,YDL(	T,T,T,T,T,T	YDL063C,Y	T,T,T,T,T,T	14433	I	Realign	Y4;P5;A2;A	MEP
chrXI	101391	111977	5	9	8.293123	4.865284	1	1	0	intron,FAS	T,F,T,T,F	YKL182W,Y	F,T,T,F	10586	I	Realign	P5;P3;A2;Y	MEP
chrXII	1071634	1072103	6	12	2085.111	797.7337	0.394434	0.351628	0	OriDB,YLR	F,F,F	YLR466C-B	F	469	I	Realign	P5;A2;Y5;A	MEP
chrI	163037	164139	0	6	1396.765	761.2807	0.332955	0.442894	0	OriDB,LTR	F,F,F	.	F	1102	V	Realign	A2	MEP
chrII	216704	221134	108	33	73.16027	14.08438	1	0.97167	0	long_term	T,F,F,T,T,F	YBL006C,Y	F,T,T	4430	V	Realign	A5	MEP
chrII	691064	701410	736	124	171.7128	37.2083	1	0.999952	0	ABD1,PRP	F,T,T,T,F,F	YBR236C,Y	F,T,T,T,F	10346	V	Realign	A4	MEP
chrIII	65514	65578	33643	1125	1263014	369551.7	0.871149	0.871149	0	RRP7	F	YCL031C	F	64	V	Realign	A5	MEP
chrIV	74492	75519	18	10	12.49951	3.066728	0.999302	1	0	PRR2	F	YDL214C	F	1027	V	Realign	A5	MEP
chrIV	538809	542807	10	9	8.566533	3.244336	1	1	0	RSM10,NR	T,F,T	YDR041W,	T,F,T	3998	V	Realign	A5	MEP
chrIV	981170	982004	2	6	635.0647	621.559	0.999648	0.451627	0	long_term	T,F,F	.	F	834	V	Realign	A5	MEP
chrIV	982598	982953	8	2	271.2507	246.9221	0.298113	0.249998	0	LTR_retrot	F,F	.	F	355	V	Realign	A5	MEP
chrIV	983380	984252	8	8	1517.667	279.6628	0.419667	0.429077	0	LTR_retrot	F,F	.	F	872	V	Realign	A5	MEP
chrIV	984128	984293	8	2	1575.176	216.0241	0.382272	0.400718	0	LTR_retrot	F,F	.	F	165	V	Realign	A5	MEP
chrIV	984498	984638	8	1	1150.379	85.3945	0.354612	0.64221	0	LTR_retrot	F,F	.	F	140	V	Realign	A5	MEP
chrIV	985522	987167	6	21	1593.528	313.8864	0.398397	0.375668	0.003541	OriDB,long	F,T,F,F,F,F	.	F	1645	V	Realign	A5	MEP
chrIV	986353	986667	6	6	2048.86	180.4864	0.254803	0.260762	0	LTR_retrot	F,F	.	F	314	V	Realign	A5	MEP
chrIV	986606	987251	6	5	1068.216	660.4424	0.372809	0.096098	0.054264	OriDB,long	F,T,F,F,F,F	.	F	645	V	Realign	A5	MEP
chrIV	992141	992642	2	5	1155.443	505.6275	0.359109	1	0	OriDB,long	F,T,F,F	.	F	501	V	Realign	A5	MEP
chrIX	308666	310340	8	20	7.757467	2.886289	1	1	0.000597	OriDB,YIL(	F,F,F	YIL024C,YI	F,F	1674	V	Realign	A5	MEP

Supplementary Table S3: List of all circular DNA recorded in the 8 yeast populations. Top 20 lines shown. Tab separated file containing the chromosomal coordinates, coverage statistics and genomic features of the detected circular DNA. Ordered from left to right, the columns contain the following information: 1,chromosome; 2, start coordinate; 3, end coordinate; 4, number of discordant read pairs; 5, number of split reads; 6, mean coverage within the detected coordinates; 7, standard deviation of the coverage within the detected coordinates; 8, read coverage ratio at the start coordinate; 9, read coverage ratio at the end coordinates; 10, fraction of reference bases not covered by sequencing reads within the detection coordinates; 11, genomic features contained on the circle; 12, True (indicated as T) and False (indicated as F) values indicating whether the genomic feature overlaps the circle at 100%; 13, ORFs contained on the circle; 14, True (indicated as T) and False (indicated as F) values indicating whether the ORFs overlaps the circle at 100%; 15, length of the circle; 16, classification of the segregation mechanism during cell division; 17, Circle-Map algorithm (Realign or Repeats) used for detection of the circle; 18, samples number identifiers(2-10) and subpopulations (A: aged, P: progeny, Y: young) were the circle was detected and 19, purification experiment were the circle was detected (MEP: Mother Enrichment Program, Biotin: Biotinylation program and Rep-MAP: replicated Mother Enrichment Program).

Gene	Motif_reg	OddsRatic	P-value	P-Adjust	In_Retain	Not_in_Re	In_lost	Not_in_lo
ABF1	[AG]TC[A(	1.5	0.5319	1	5	6	70	126
ACE2	ACCAGC	1.6337	0.5355	1	6	5	83	113
ADR1	G[AG]GG	0.4	0.2458	1	9	2	180	16
AFT1	[TC][AG]C	1.9219	0.4017	1	3	8	32	164
ARO80	CGG[GAT(	2.1595	0.3816	1	3	8	29	167
ASH1	[AT][AT][(	1.0402	1	1	8	3	141	55
AZF1	AAGAAAA	1.6795	0.514	1	5	6	65	131
BAS1	TGACTC	1.6414	0.5166	1	5	6	66	130
YAP1	TTAGT[CA	1.3269	0.7387	1	4	7	59	137
CAT8	CC[AG][G/	0.9145	1	1	3	8	57	139
CBF1	TCACGTG	0.88	1	1	1	10	20	176
CHA4	CGG[GAT(	2.0332	0.2753	1	4	7	43	153
CRZ1	G[GATC]G	1.52	0.6397	1	2	9	25	171
CUP2	[ACT]T[A(	2.5083	0.2214	1	8	3	101	95
GATA	GATAAG	1.0019	1	1	5	6	89	107
ECM22/UF	TATACGA	0	0.3765	1	0	11	31	165
FKH1	[GA]TAAA	7.0449	0.0029	0.229	7	4	39	157
FLO8	AAAACCT	0	1	1	0	11	0	196
FZF1	CGTATCGT	0	1	1	0	11	0	196

Supplementary Table S4: Top 20 lines shown. Tab separated file containing the *cis* acting element binding sites tested for overrepresentation between circular DNA classified as lost and circular DNA classified as retained (class I and II). Ordered from left to right, the columns contain the following information: 1, *cis* acting element name tested; 2, regular expression used for searching the *cis* acting element in the reference sequence; 3, Fisher's exact test odds ratio; 4, Fisher's exact test *p-value*; 5, adjusted *p-value*; 6, number of circular DNA containing the *cis* acting element in the retained group; 7, number of circular DNA not containing the *cis* acting element in the retained group; 8, number of circular DNA containing the *cis* acting element in the lost group and 9, number of circular DNA not containing the *cis* acting element in the lost group.

Gene	Motif_regi	OddsRatio	P-value	P-Adjust	In_Retaine	Not_in_Re	In_lost	Not_in_los
OriDB	.	9,2121	0,0009	0,0062	8	3	44	152
centromer	.	NaN	1	1	0	0	0	0
long_term	.	1,4077	0,5463	0,956	1	10	13	183
telomere	.	43,3333	0,0075	0,0263	2	9	1	195
LTR_retrot	.	2,35	0,3944	0,9202	1	10	8	188
intron	.	0,9889	1	1	1	10	18	178
Repetitive	.	0	0,013	0,0907	6	5	3	193

Supplementary Table S5: Tab separated file containing the genomic features tested for overrepresentation between circular DNA classified as lost and circular DNA classified as retained (class I and II). Ordered from left to right, the columns contain the following information: 1, genomic feature tested; 2, Fisher's exact test odds ratio; 3, Fisher's exact test *p-value*; 4, adjusted *p-value*; 5, number of circular DNA containing the genomic feature in the retained group; 6, number of circular DNA not containing the genomic feature in the retained group; 7, number of circular DNA containing the genomic feature in the lost group and 8, number of circular DNA not containing the genomic feature in the lost group.

circle	Y2	Y3	Y4	Y5	A2	A3	A4	A5	P2	P3	P4	P5
chrIV 5303	4.251054	1081.488	0	1.60195	0	5.332839	13.4748	27.50539	2326.559	528.2875	1592.676	486.1212
chrIV 1154	68.01686	101.6675	7986.136	6.407798	13.22455	0	159.4518	3.056154	3401.734	11.80531	13.67104	63.72375
chrVIII 212	69.23145	1721.993	13.51292	28.83509	50.2533	10.66568	0	6.112308	206.0752	123.9557	391.9031	9.103393
chrXII 4514	427040.8	1296180	2510984	716504	3446361	4433656	3951704	3812265	3346273	80160.99	3583935	275826.6
chrIII 8298	43.72513	0	0	0	0	0	2.245801	0	540.5741	0	0	4.005493
chrIV 9572	64.98039	382.5239	173.4158	509551.3	111.0862	141.3202	103.3068	100.8531	140.3701	132.8097	141.2674	33.50049
chrIV 1118	94.73777	669.7345	279.267	956969.4	187.7886	322.6368	312.1663	275.0539	367.3514	525.3362	250.6357	87.39258
chrIV 3382	652.2331	3135760	1450.387	1100.539	1195.5	1517.193	1291.335	1470.01	1406.687	1744.234	1291.913	234.5034
chrIV 8777	2.429174	2.541687	0	201.8456	60.83294	2.666419	11.229	6.112308	4252.914	5.902654	2.278506	1666.649
chrVII 852	96.55965	0	0	0	0	0	0	0	0	0	0	14.20129
chrX 6406	315.7926	1342.011	635.1073	2094115	409.9611	573.2802	642.299	586.7816	668.9977	519.4336	524.0564	123.442
chrXI 1013	898.187	1776.639	13363427	2390.109	2890.887	2917.063	2775.809	2860.56	3539.118	2263.668	2927.88	476.6537
chrXII 107	5581.634	17671.08	693.6633	20700.39	17035.87	23731.13	13512.98	29363.53	1711.32	97.39379	15204.47	2469.933

Supplementary Table S6: Tab separated file containing the median of the ratios normalized values for the circular DNA detected in at least two samples of the Mother Enrichment Program experiment. Ordered from left to right, the columns contain the following information: 1, circular DNA detection coordinates together with the number of samples it was detected; 2-13, sequencing samples (Y indicates the young population, A indicates the aged population and P indicates the progeny population).

sample	rdna	phenotype
Y10	3.4244	young
Y6	0.2659	young
Y7	23.016	young
Y9	1.6526	young
A10	68.576	aged
A6	491.19	aged
A7	142.11	aged
A9	1395.7	aged

Supplementary Table S7: Tab separated file containing the plasmid normalized read counts for circular DNA formed from the rDNA locus. Ordered from left to right, the columns contain the following information: 1, sample (Y indicates young and A indicates aged); 2, plasmid normalized read count values; 3: phenotype of the sample.

sample	y_prime	phenotype
Y10	0.003225	young
Y6	2.89E-05	young
Y7	0.023299	young
Y9	8.70E-05	young
A10	0.046857	aged
A6	0.494467	aged
A7	0.127192	aged
A9	1.116935	aged

Supplementary Table S8: Tab separated file containing the plasmid normalized read counts for circular DNA formed from the Y’ prime telomeric locus. Ordered from left to right, the columns contain the following information: 1, sample (Y indicates young and A indicates aged); 2, plasmid normalized read count values; 3: phenotype of the sample.

circle	young	aged	sample
chrII:246815-248139	5.93E+10	0	Y10
chrII:397991-399258	0.000107638	0	Y10
chrIII:132810-134416	0.000244136	0	Y10
chrIV:337585-338119	0.000148977	0.000713542	Y10
chrIV:1168614-116990	0.000102178	0	Y10
chrVII:450415-451370	0.000116218	0	Y10
chrXI:550390-553603	0.000713688	0	Y10
chrXIII:134428-135524	0.000404033	0	Y10
chrXV:24081-24630	2.81E+11	0	Y10
chrXV:942397-943448	3.82E+11	0	Y10
chrII:362003-363899	4.73E+10	0	Y6
chrII:365121-365650	1.09E+11	0	Y6
chrII:370337-370696	4.79E+09	0	Y6
chrII:594391-594670	2.18E+11	0.859587492	Y6
chrII:716051-716401	0.001515229	1.36E+11	Y6
chrIII:43409-55194	0.00021835	1.19E+10	Y6
chrIII:75328-75660	3.14E+09	0	Y6
chrIV:105879-106282	2.68E+10	0	Y6
chrIV:215091-225427	0.000225127	0	Y6

Supplementary Table S9: Top 20 lines shown. Tab separated file containing the plasmid normalized read counts for all the circular DNA from other parts of the genome, excluding the rDNA and the Y' telomeric locus. Ordered from left to right, the columns contain the following information: 1, circular DNA detection chromosomal coordinates; 2, plasmid normalized read count values in the young sample; 3, plasmid normalized read count values in the aged sample, Young sample where the circular DNA was detected.

Supplementary files

Supplementary file S1. FASTA formatted file containing the DNA sequence of the circular DNA formed from the [*Y' telomeric* ^{circle}] locus.

Supplementary Figure S1

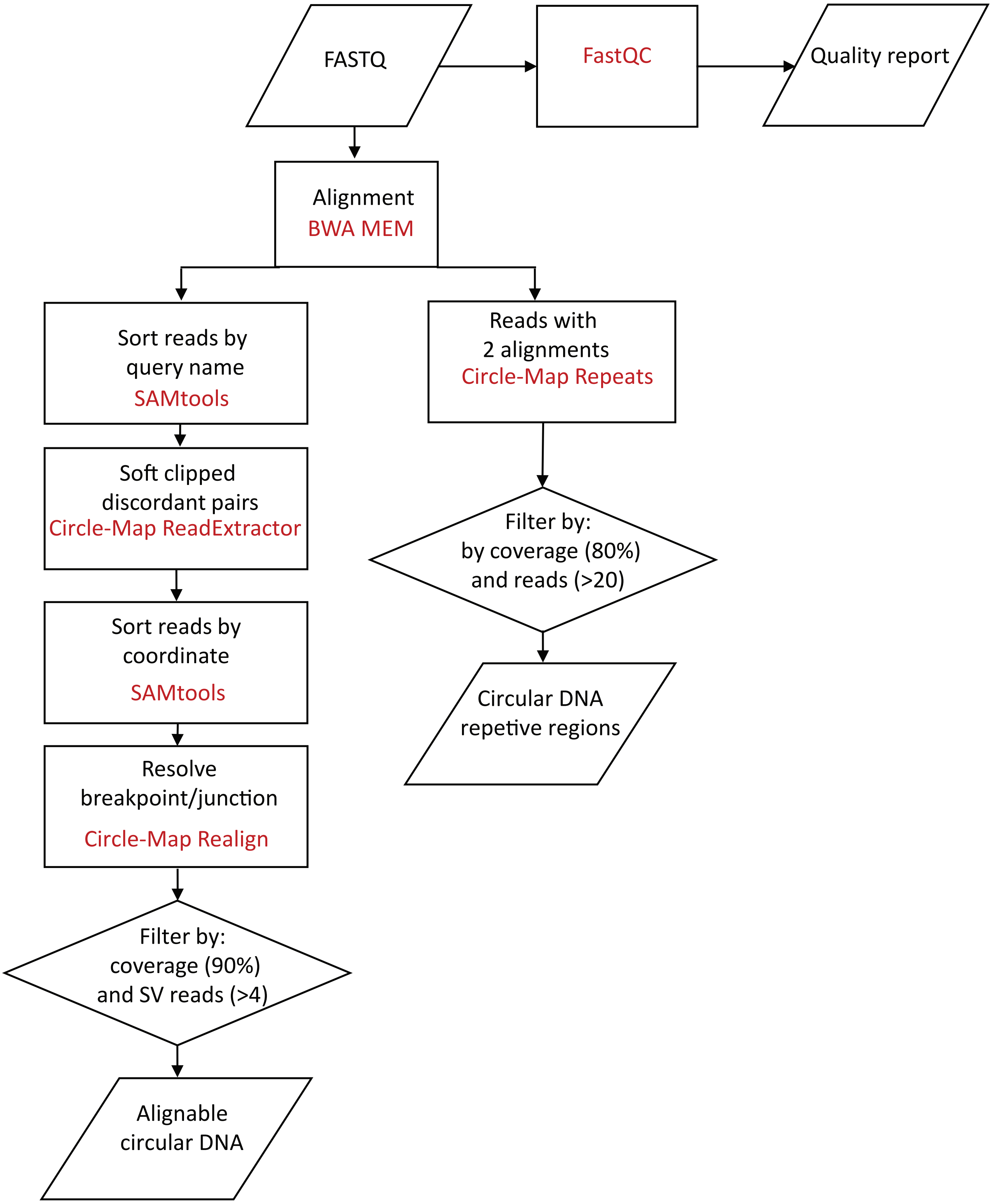

Suplementary figure S2

A

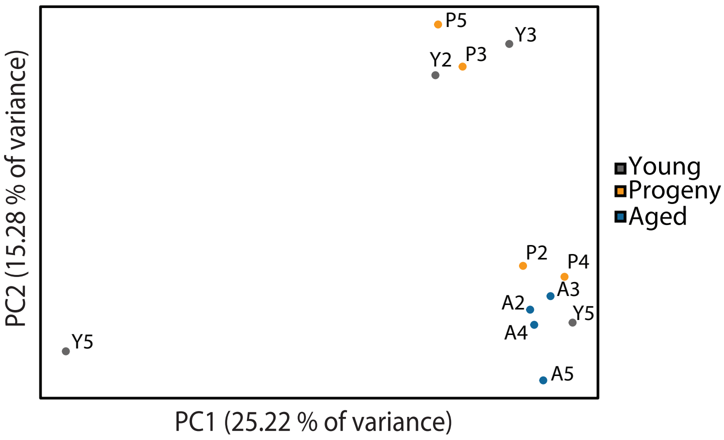

Supplementary Figure S3

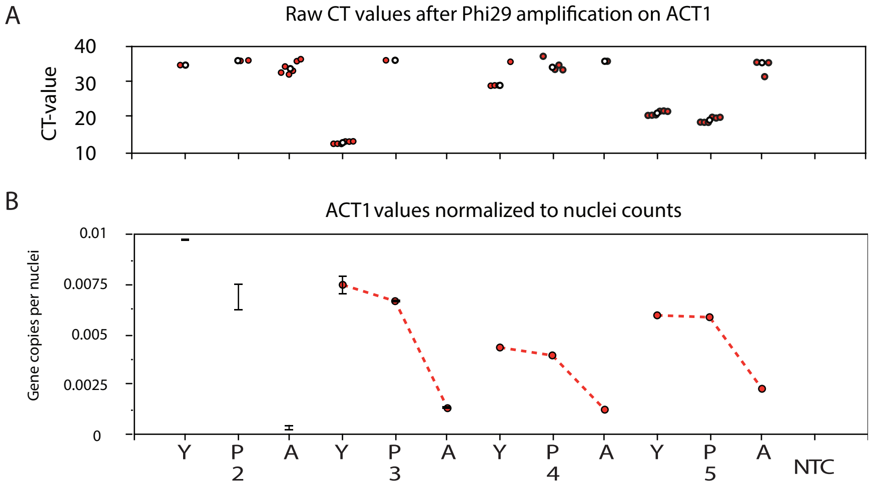

Supplementary figure S4

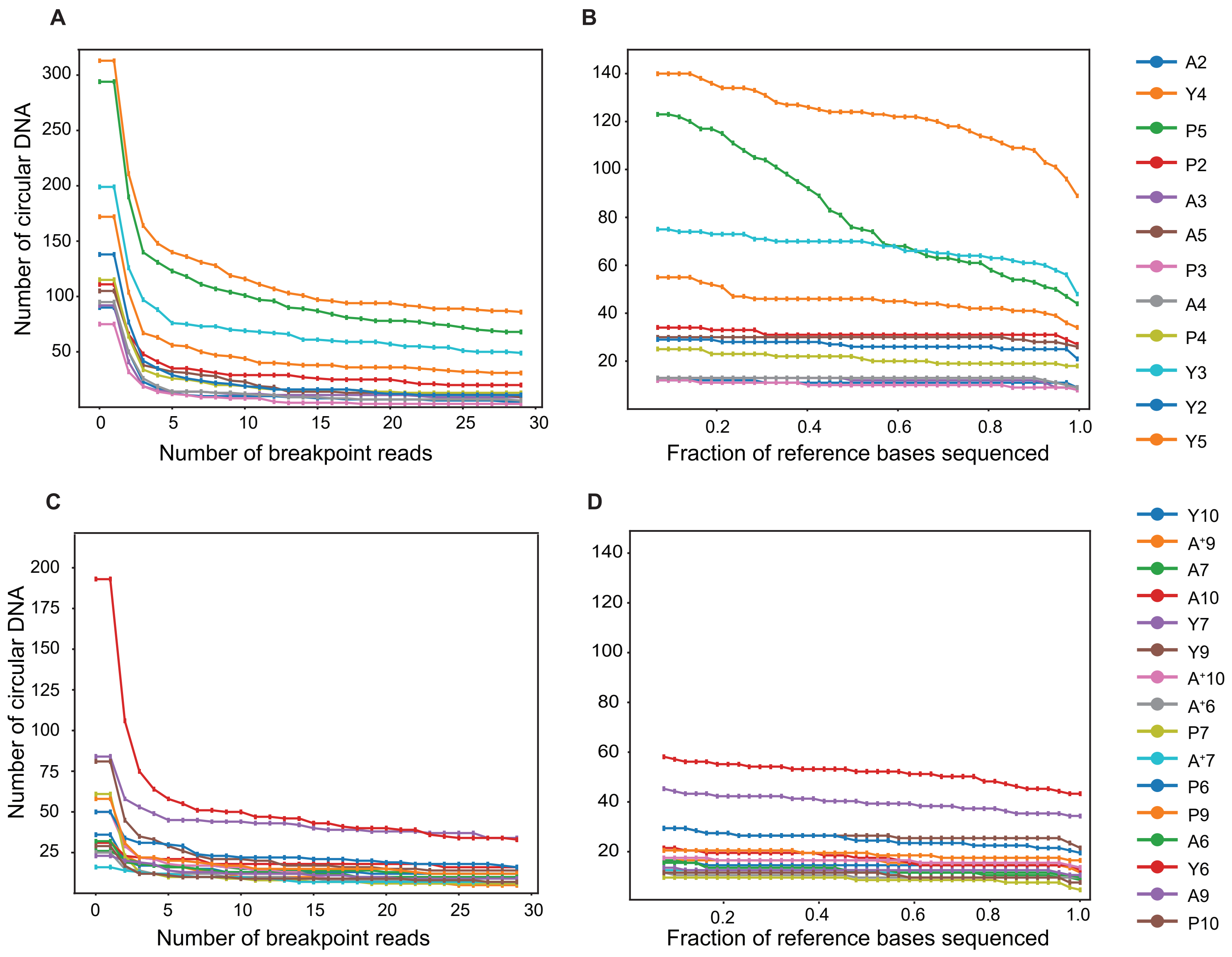

Supplementary figure S5

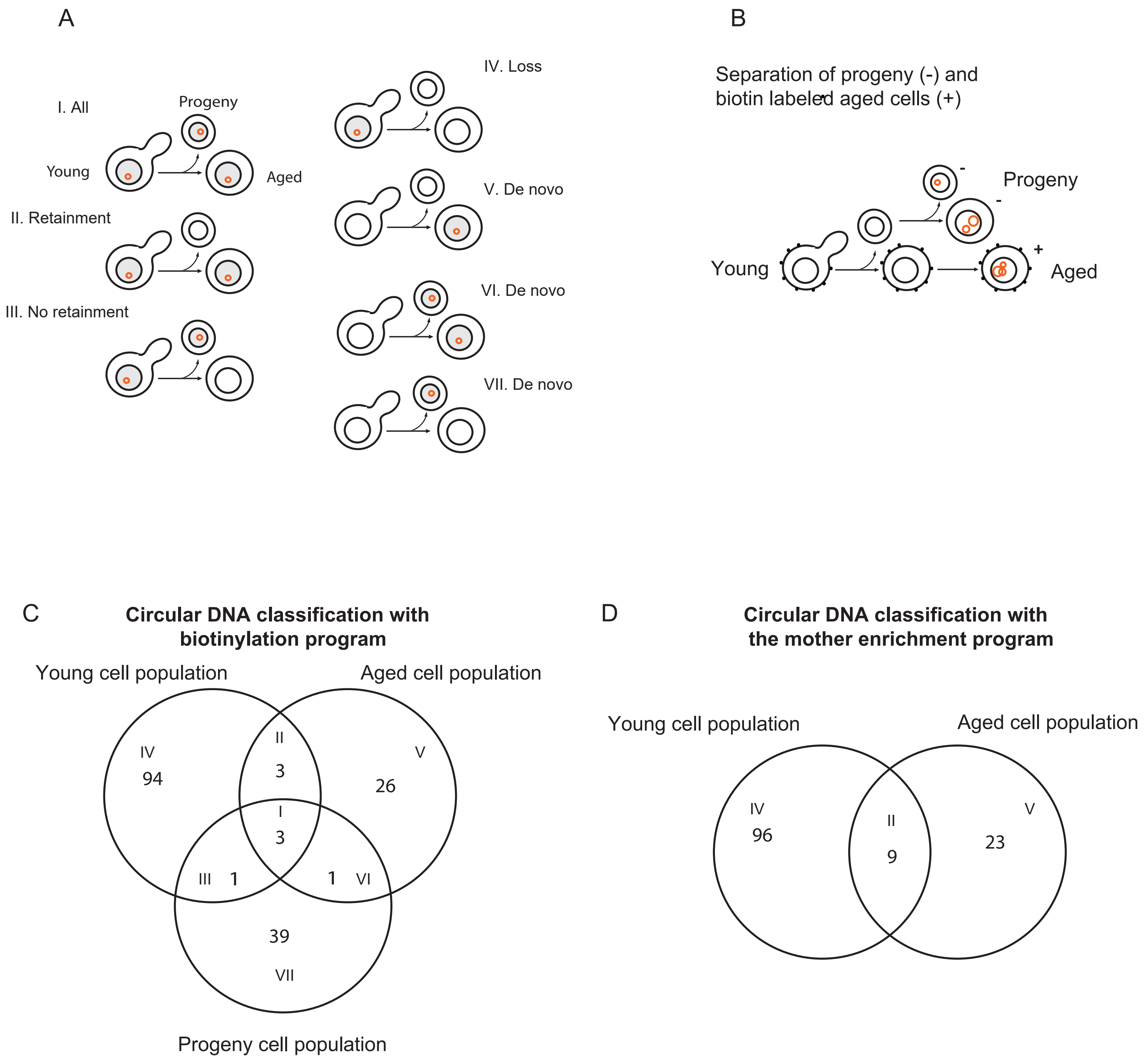

Supplementary figure S6

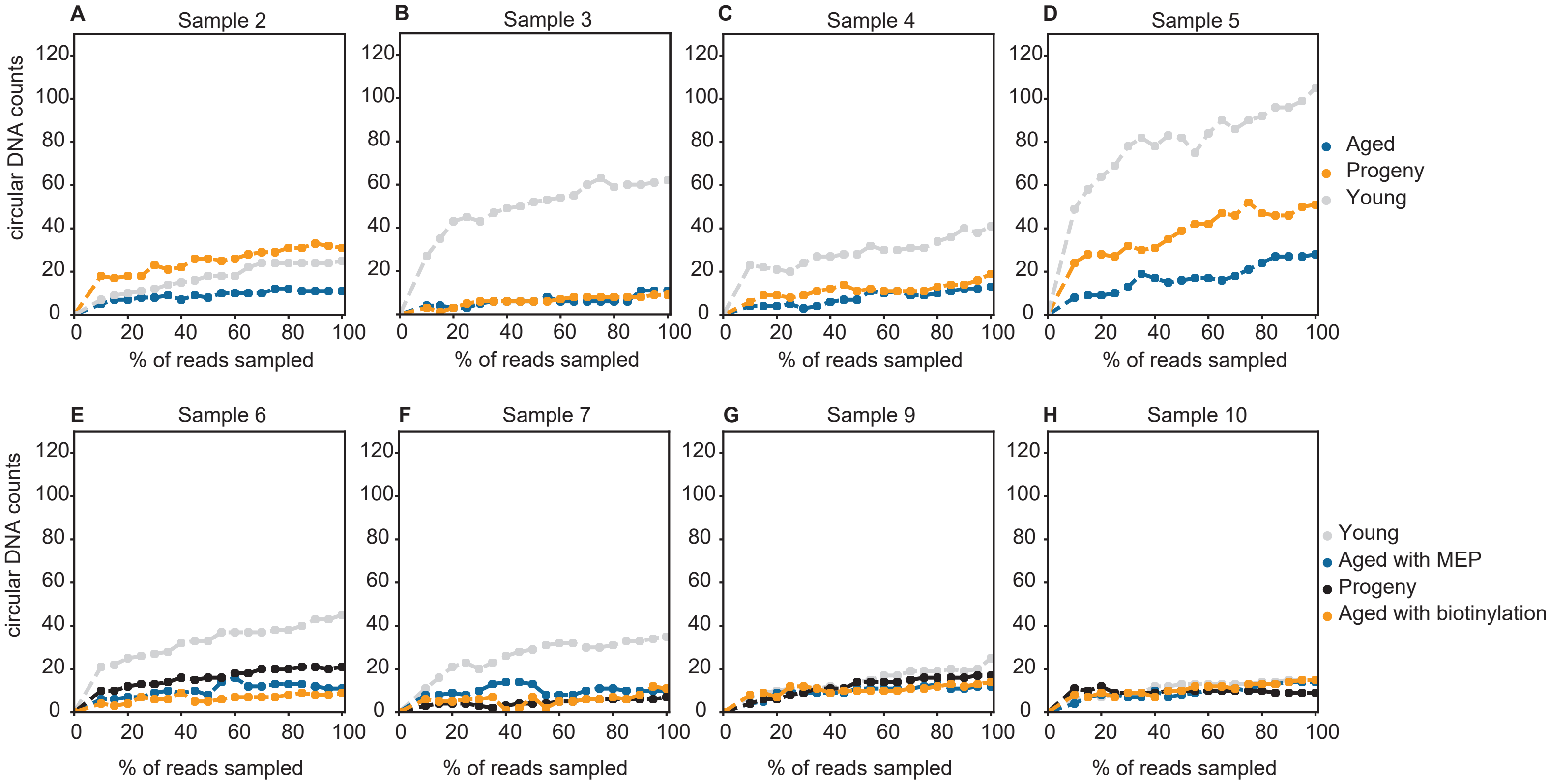

Supplementary figure S7

A

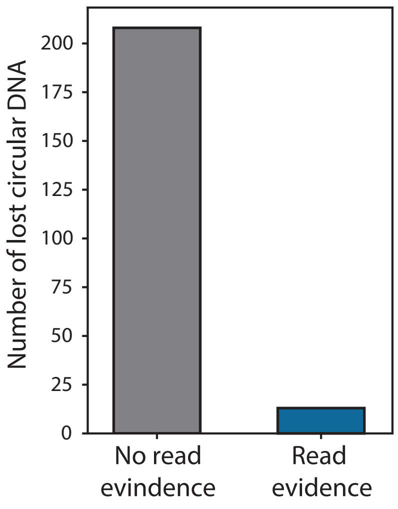

Supplementary figure S8

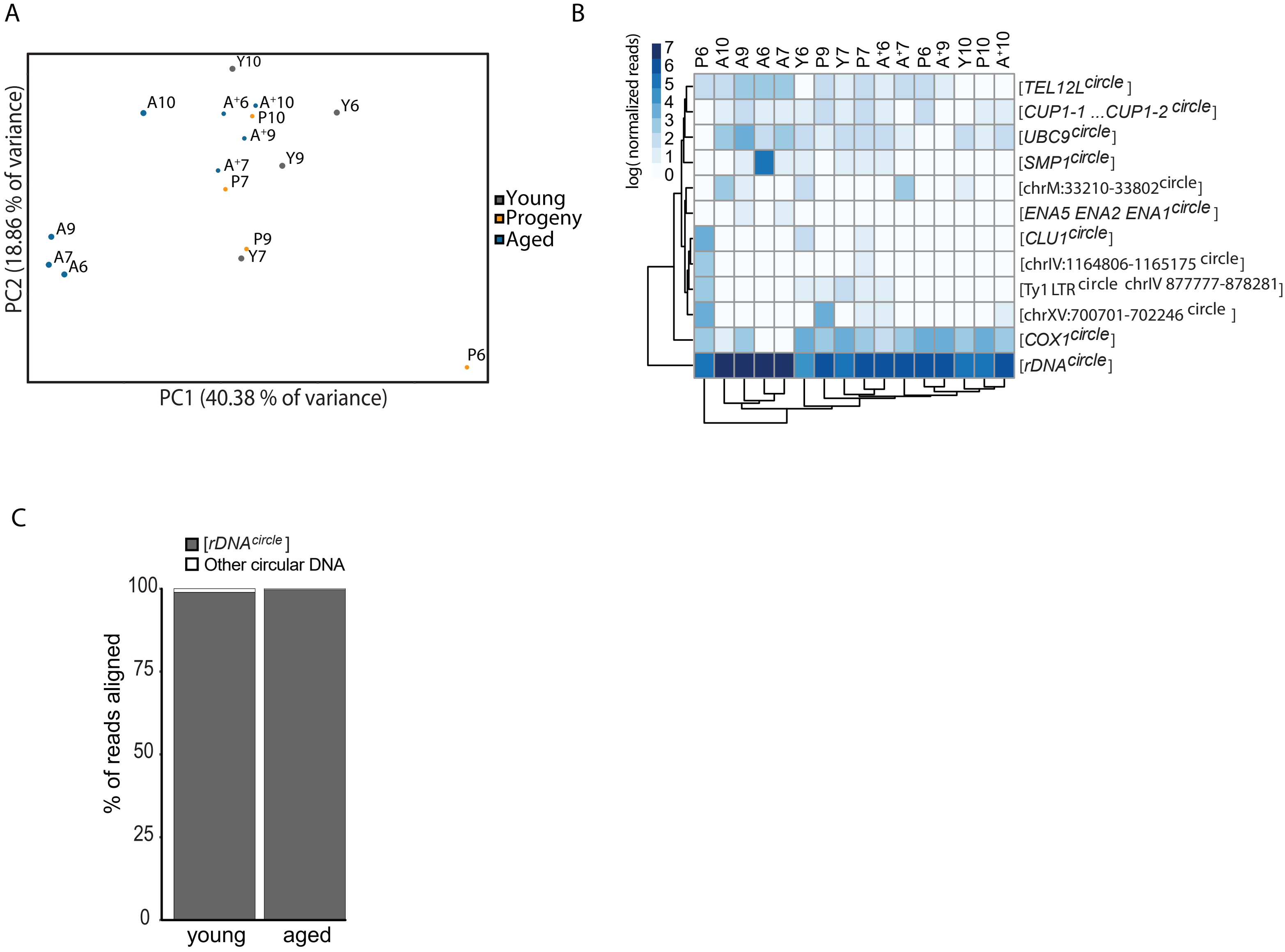

Supplementary figure S9

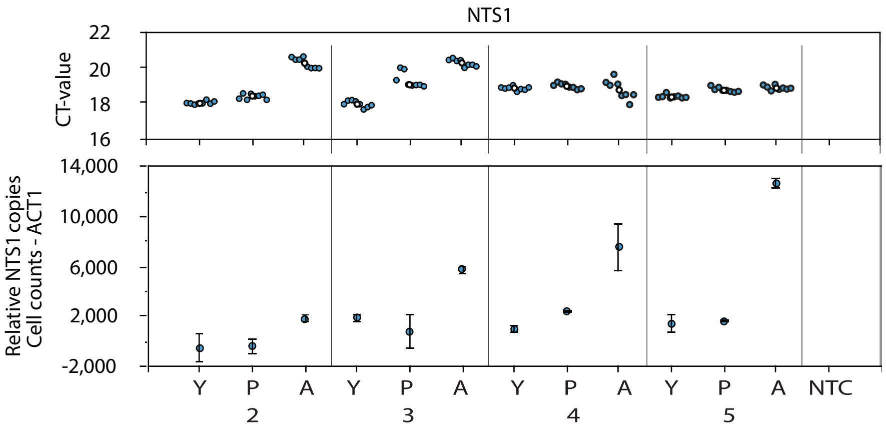

Supplementary figure S10

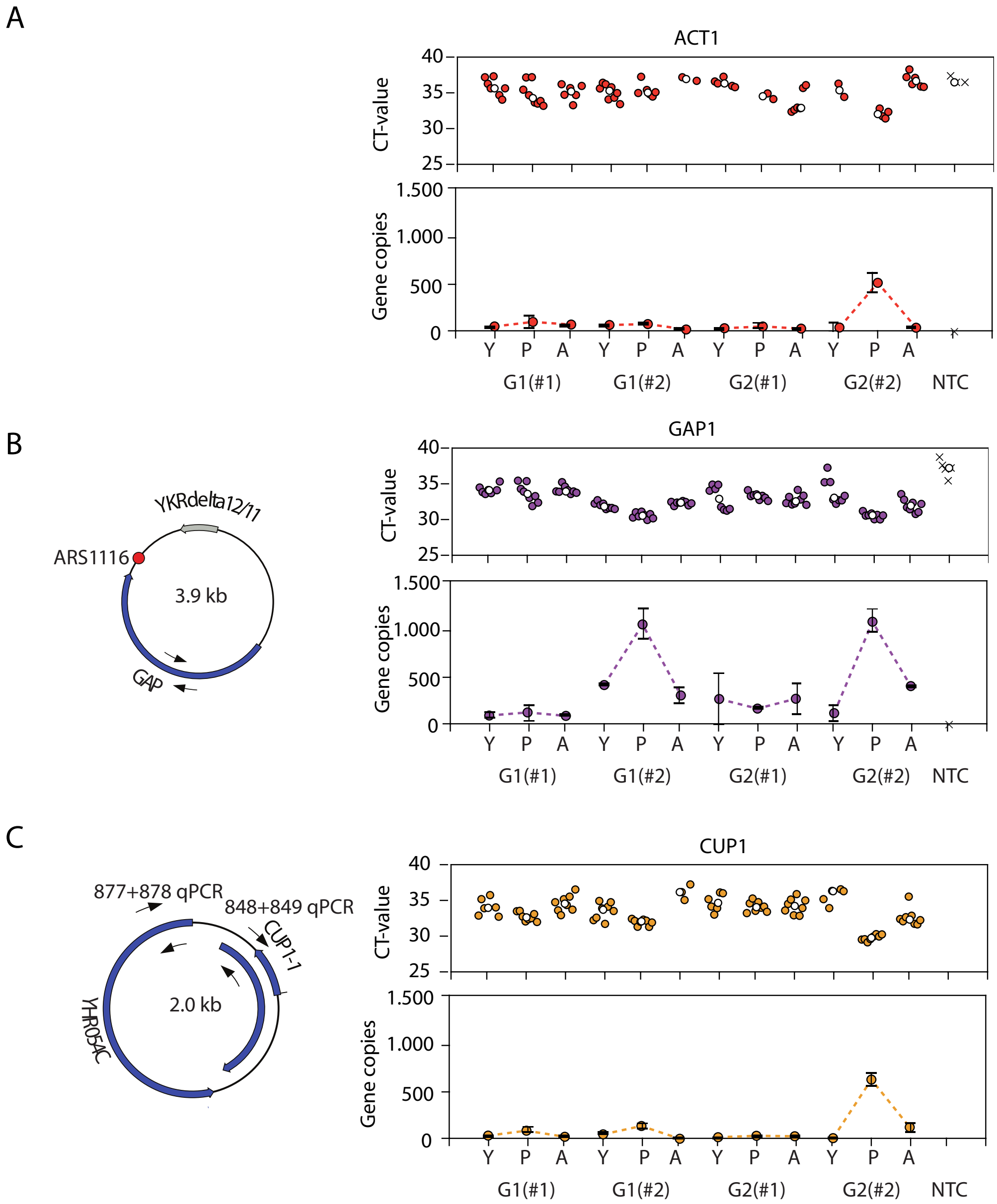

Supplement: gkaa545_Supplemental_Files [file gkaa545_supplemental_files.zip › supplements_merged.pdf]
